# Predicting Directions of Changes in Genotype Proportions Between Norovirus Seasons in Japan

**DOI:** 10.3389/fmicb.2019.00116

**Published:** 2019-02-05

**Authors:** Yoshiyuki Suzuki, Yen Hai Doan, Hirokazu Kimura, Hiroto Shinomiya, Komei Shirabe, Kazuhiko Katayama

**Affiliations:** ^1^Graduate School of Natural Sciences, Nagoya City University, Nagoya, Japan; ^2^Department of Virology II, National Institute of Infectious Diseases, Musashimurayama, Japan; ^3^Graduate School of Health Science, Gunma Paz University, Takasaki, Japan; ^4^Department of Microbiology, Ehime Prefecctural Institute of Public Health and Environmental Science, Matsuyama, Japan; ^5^Division of Virology, Yamaguchi Prefectural Institute of Public Health and Environment, Yamaguchi, Japan; ^6^Kitasato Institute for Life Sciences, Kitasato University, Minato, Japan

**Keywords:** genotype proportion, herd immunity, norovirus, prediction, vaccine

## Abstract

The norovirus forecasting system (NOROCAST) has been developed for predicting directions of changes in genotype proportions between human norovirus (HuNoV) seasons in Japan through modeling herd immunity to structural protein 1 (VP1). Here 404 nearly complete genomic sequences of HuNoV were analyzed to examine whether the performance of NOROCAST could be improved by modeling herd immunity to VP2 and non-structural proteins (NS) in addition to VP1. It was found that the applicability of NOROCAST may be extended by compensating for unavailable sequence data and observed genotype proportions of 0 in each season. Incorporation of herd immunity to VP2 and NS did not appear to improve the performance of NOROCAST, suggesting that VP1 may be a suitable target of vaccines.

## Introduction

Norovirus (NoV) is an etiological agent of acute gastroenteritis in humans (Kapikian et al., 1972), causing 18% of all cases (Ahmed et al., 2014) and 200,000 deaths worldwide annually (Patel et al., 2008). The human NoV (HuNoV) is more prevalent in developed countries (20%) and low-mortality developing countries (19%) than in high-mortality developing countries (14%) (Ahmed et al., 2014). Therefore, improvement in sanitation and hygiene may not be sufficient for control and prevention of HuNoV, and development of vaccines is demanded.

HuNoV belongs to the genus *Norovirus* in the family *Caliciviridae* (Clarke et al., 2012). The virion of HuNoV is non-enveloped, icosahedral, and 38 nm in diameter (Prasad et al., 1999). The genome of HuNoV is a linear, non-segmented, single-stranded, positive-sense RNA of 7.5 kb, containing ORF1-ORF3 (Jiang et al., 1993; Lambden et al., 1993). ORF1 encodes non-structural proteins (NS), whereas ORF2 and ORF3 encode structural proteins 1 (VP1) and 2 (VP2), respectively.

Based on the similarity in the amino acid sequence of VP1, NoV is divided into genogroups GI-GVII (Zheng et al., 2006; Vinje, 2015). HuNoV consists of GI, GII, and GIV strains, which are further classified into genotypes GI.1-GI.9, GII.1-GII.22, and GIV.1 and GIV.2, respectively (Kroneman et al., 2013; Vinje, 2015). Multiple genotypes of HuNoV co-circulate every season changing genotype proportions (Suzuki et al., 2016; Thongprachum et al., 2016).

Different genotypes of HuNoV appeared to be antigenically related to each other with various degrees (Hansman et al., 2006) and have been grouped into several immunotypes (Parra et al., 2017). In addition, the HuNoV infection appeared to be mediated via person-to-person, food-borne, and water-borne transmissions with some preferences depending on the genotypes (Mathijs et al., 2012). It may therefore be useful to predict changes in genotype proportions between HuNoV seasons for determining the genotypes to be included in vaccines and the human populations to be vaccinated in each season.

The norovirus forecasting system (NOROCAST) has been developed for predicting changes in genotype proportions between HuNoV seasons in Japan (Suzuki et al., 2016). The herd immunity to VP1 was taken into account based on the fitness model that was originally proposed for describing changes in proportions of influenza A virus strains (Luksza and Lassig, 2014; Suzuki, 2015). The purpose of the present study was to examine whether the performance of NOROCAST could be improved by taking into account the herd immunity to VP2 and NS in addition to VP1.

## Method

In Japan, the HuNoV season is defined such that season 2006/2007 represents the period between September of 2006 and August of 2007. Genotype frequencies in HuNoV seasons in Japan have been surveyed by the National Institute of Infectious Diseases (NIID), Japan, and deposited in the Infectious Agents Surveillance Report (IASR). The observed genotype frequencies were available for the analysis from season 2006/2007 to season 2016/2017 (Supplementary Table S1), which were converted into the observed genotype proportions (Supplementary Table S2). Multiple genotypes of GI and GII strains but no GIV strains were observed in these seasons.

Nearly complete genomic sequences of GI and GII strains containing the entire coding regions of VP1, VP2, and NS provided with the information on the isolation year and month, were retrieved from the International Nucleotide Sequence Database (INSD). The INSD accession numbers for the 404 sequences analyzed in the present study were listed in Supplementary Table S3. These sequences were classified according to the isolation season and the VP1 genotype, which was determined by the Norovirus Genotyping Tool (version 1.0) (Kroneman et al., 2011) (Supplementary Table S4). It appeared that the sequence data were biased toward more prevalent genotypes and were sometimes unavailable for less prevalent genotypes in each season. For each of VP1, VP2, and NS, multiple alignment of 404 amino acid sequences was made using the computer program MAFFT (version 7.215) (Katoh et al., 2002).

In the fitness model (Luksza and Lassig, 2014), the proportion of strain *i* in the target season *t* (*p*_*i*(*t*)_) was predicted from that in the season immediately before the target season (pre-target season) *t* − 1 (*p*_*i*(*t*−1)_) by

(1) $$\begin{array}{r} {\hat{p}}_{i\left(t\right)}=p_{i\left(t-1\right)}\times e^{f_{i}}, \end{array}$$

where *f*_*i*_ is the fitness of strain *i*. In NOROCAST (Suzuki et al., 2016), *f*_*i*_ was defined as

(2) $$\begin{array}{r} f_{i}=f_{0}-c_{i}, \end{array}$$

where *f*_0_ is a constant to ensure $\underset{i}{\sum }{\hat{p}}_{i\left(t\right)}=1$. The term *c*_*i*_ denotes the effect of herd immunity to VP1 of strain *i* elicited by strain *j*'s that have circulated from season 2006/2007 to the pre-target season, namely

(3) $$\begin{array}{r} c_{i}=\sum_{j}k_{\text{VP}1}\times {\left(1-l_{\text{VP}1}\right)}^{d_{ij\left(\text{VP}1\right)}}\times 0.8^{t_{i}-t_{j}+1}\times p_{j\left(t_{j}\right)}. \end{array}$$

Here *k*_VP1_ is the coefficient of herd immunity to VP1, which was assumed to decline by fraction *l*_VP1_ for each of amino acid differences in VP1 between strains *i* and *j* designated as *d*_*ij*(*VP*1)_ (Lindesmith et al., 2011). The terms *t*_*i*_ and *t*_*j*_ denote the isolation seasons of strains *i* and *j*, respectively. The herd immunity was assumed to decline 20% each season, so that the expected duration of human immunity was roughly 5 years (Simmons et al., 2013).

In the present study, the herd immunity to VP2 and NS was also taken into account, so that *c*_*i*_ was re-defined as

(4) $$\begin{array}{l} c_{i}=\sum_{j}[k_{\text{VP1}}\times {\left(1-l_{\text{VP1}}\right)}^{d_{ij(\text{VP1})}}+k_{\text{VP2}}\times {\left(1-l_{\text{VP2}}\right)}^{d_{ij(\text{VP2})}}\\ \;+k_{\text{NS}}\times {\left(1-l_{\text{NS}}\right)}^{d_{ij(\text{NS})}}]\times 0.8^{t_{i}-t_{j}+1}\times p_{j\left(t_{j}\right)}. \end{array}$$

Here *k*_VP2_, *l*_VP2_, and *d*_*ij*(VP2)_ and *k*_NS_, *l*_NS_, and *d*_*ij*(NS)_ are parameters for VP2 and NS, respectively, corresponding to *k*_VP1_, *l*_VP1_, and *d*_*ij*(VP1)_ for VP1, respectively. For predicting genotype proportions in the target season, parameters were optimized using the observed genotype proportions from season 2006/2007 to the pre-target season. Optimization was performed with the genetic algorithm (Tomita et al., 2000) through minimizing the sum of squared deviations of the predicted genotype proportions from the observed genotype proportions over the seasons from season 2008/2009 to the pre-target season. Three random real numbers were assigned as the initial parameter values to confirm the convergence of the optimization (data not shown) (Suzuki, 2013, 2015). The optimized parameter values were adopted for predicting directions of changes in genotype proportions from the pre-target season to the target season.

The performance of NOROCAST was evaluated retrospectively by predicting directions of changes in genotype proportions setting the target seasons to be from season 2008/2009 to season 2016/2017. It should be noted that in NOROCAST (Suzuki et al., 2016), genotype proportions could not be predicted or were always predicted to be 0 when the sequence data were unavailable or the observed genotype proportions were 0 in the pre-target season, respectively. Therefore, the prediction was considered to be correct when both the observed and the predicted directions of changes in genotype proportions (increase or decrease) were the same, and incorrect when they were different. In the present study, unavailable sequence data in a particular season were compensated by the available sequence data in the closest season before or after that season or both within the same genotype (Supplementary Table S5). The observed genotype proportions of 0 were also replaced with an arbitrary value (5 × 10^−6^, 5 × 10^−5^, or 5 × 10^−4^) that was smaller than 5.099 × 10^−4^ ($=\frac{1}{1961}$), which was the lowest positive value obtainable in Supplementary Table S2.

## Results

The accuracy of NOROCAST in predicting directions of changes in genotype proportions was examined setting the target seasons to be from season 2008/2009 to season 2016/2017 (Supplementary Tables S6–S14). When only the herd immunity to VP1 was taken into account without replacing the observed genotype proportions of 0, the numbers of correct and incorrect predictions were 66 and 62, respectively, yielding the accuracy of 0.516 (*P* = 0.724 by the χ^2^ test) (Table 1). However, by replacing the observed genotype proportions of 0 with 5 × 10^−6^, 5 × 10^−5^, or 5 × 10^−4^, directions were predicted correctly and incorrectly in 86 and 62 cases, respectively, so that the accuracy was 0.581 (*P* = 0.0485 by the χ^2^ test). The accuracy was elevated mainly because changes in genotype proportions became predictable even when the observed genotype proportions were 0 in the pre-target season (Table 1).

**Table 1:** Numbers of observed and predicted directions of changes in genotype proportions setting the target seasons to be from season 2008/2009 to season 2016/2017.

**Protein**	**Arbitrary value**	**Predicted**	**Observed**	
			**Increase**	**Decrease**
VP1	0	Increase	51	59
		Decrease	3	15
	5 × 10^−6^	Increase	71	59
		Decrease	3	15
	5 × 10^−5^	Increase	71	59
		Decrease	3	15
	5 × 10^−4^	Increase	71	59
		Decrease	3	15
VP1, VP2, and NS	0	Increase	49	59
		Decrease	5	15
	5 × 10^−6^	Increase	69	58
		Decrease	5	16
	5 × 10^−5^	Increase	70	58
		Decrease	4	16
	5 × 10^−4^	Increase	71	58
		Decrease	3	16

However, the accuracy of NOROCAST did not appear to be improved by taking into account the herd immunity to VP2 and NS in addition to VP1 (Supplementary Tables S6–S14). That is, without replacing the observed genotype proportions of 0, both the numbers of correct and incorrect predictions were 64, yielding the accuracy of 0.5 (*P* = 1 by the χ^2^ test) (Table 1). By replacing the observed genotype proportions of 0 with 5 × 10^−6^, 5 × 10^−5^, or 5 × 10^−4^, directions were predicted correctly and incorrectly in 85–87 and 61–63 cases, respectively, so that the accuracy was 0.574–0.588 (*P* = 0.0326–0.0705 by the χ^2^ test) (Table 1).

It should be noted that the direction of change in genotype proportion was always (9 out of 9 cases with VP1; *P* = 0.00391 by the binomial test) or almost always (8 out of 9 cases with VP1, VP2, and NS; *P* = 0.0352 by the binomial test) predicted correctly for GII.4, which was the most prevalent genotype in HuNoV (Supplementary Tables S6–S14). Interestingly, GII.4 has been classified as the evolving genotype, whereas other genotypes have been classified as the static genotypes (Parra et al., 2017). It is possible that GII.4 strains may be influenced by the herd immunity more strongly than the strains of other genotypes, which may have resulted in faster evolution and higher predictability of the direction of change in genotype proportion for GII.4 than other genotypes.

## Discussion

In the previous study (Suzuki et al., 2016), NOROCAST was developed for predicting directions of changes in genotype proportions between HuNoV seasons in Japan modeling the herd immunity to VP1. From the analysis of 1458 VP1 sequences setting the target seasons to be from season 2008/2009 to season 2014/2015, directions were predicted correctly and incorrectly in 45 and 30 cases, respectively, yielding the accuracy of 0.6 (*P* = 0.0833 by the χ^2^ test). In the present study, the effect of modeling herd immunity to VP2 and NS in addition to VP1 was examined from the analysis of 404 nearly complete genomic sequences of HuNoV.

When only the herd immunity to VP1 was taken into account without replacing the observed genotype proportions of 0, directions were predicted correctly and incorrectly in 52 and 48 cases, respectively, for the same target seasons as above, so that the accuracy was 0.52 (*P* = 0.689 by the χ^2^ test). Compared with the previous results, the number of predictions was raised because unavailable sequence data were compensated but the accuracy was lowered possibly because a smaller number of sequences were analyzed in the present study. However, the accuracy was recovered by replacing the observed genotype proportions of 0 with an arbitrary value that was smaller than the lowest positive value obtainable as the observed genotype proportion over all HuNoV seasons.

In contrast, the performance of NOROCAST did not appear to be improved by incorporation of herd immunity to VP2 and NS, suggesting that VP1-derived virus-like particle (VLP) and VP1-P domain-derived P-particle may be suitable as vaccines (Hansman et al., 2006; Kocher and Yuan, 2015). Nevertheless, VP2 and NS may still be incorporated into the fitness model for improving the performance of NOROCAST. It may be interesting to model the fitness effects of environmental conditions such as temperature, humidity, and pH, which may affect the functions of VP2 and NS as well as VP1 (de la Noue et al., 2014; Samandoulgou et al., 2015). In addition, the fitness effect of herd immunity to VP1 may depend not only on the number of amino acid differences between HuNoV strains but also on their impacts on the binding affinity to the HuNoV receptor, which has not been identified yet (Murakami et al., 2013; Haga et al., 2016; Orchard et al., 2016). It may be critical to identify the amino acid sites in VP1, VP2, and NS involved in these functions.

When the target season was set to be season 2017/2018, it was consistently predicted that the proportions of GII.2-GII.4 and GII.17 may decrease, whereas those of other genotypes may increase, regardless of whether the observed genotype proportions of 0 were replaced with 5 × 10^−6^, 5 × 10^−5^, or 5 × 10^−4^ and whether the herd immunity to VP2 and NS was taken into account in addition to VP1 (Supplementary Table S15). The predicted genotype proportions can be seen on the NOROCAST website (URL: http://www.nsc.nagoya-cu.ac.jp/~yossuzuk/norocast.html). To validate these results and predict genotype proportions in the future HuNoV seasons, it may be critical to update the observed genotype frequencies and the sequence data of HuNoV.

## Author Contributions

YS, YHD, HK, HS, KS, and KK designed this study and wrote the manuscript. YS and YHD analyzed the data. HK, HS, KS, and KK supervised this study. All authors read and approved the manuscript.

### Conflict of Interest Statement

The authors declare that the research was conducted in the absence of any commercial or financial relationships that could be construed as a potential conflict of interest.
