# Supplementary material for: Predicting Directions of Changes in Genotype Proportions Between Norovirus Seasons in Japan
Source: Front Microbiol. 2019 Feb 5;10:116. doi: 10.3389/fmicb.2019.00116 (PMC6370659; doi:10.3389/fmicb.2019.00116)
Supplement: Supplementary file 1 [file Table_1.DOCX]

**Supplementary table S1:** Observed genotype frequencies of HuNoV strains in Japan from season 2006/2007 to season 2016/2017 deposited in IASR.

| Genotype | 2006/2007 | 2007/2008 | 2008/2009 | 2009/2010 | 2010/2011 | 2011/2012 | 2012/2013 | 2013/2014 | 2014/2015 | 2015/2016 | 2016/2017 |
| --- | --- | --- | --- | --- | --- | --- | --- | --- | --- | --- | --- |
| GI.1 | 1 | 4 | 1 | 3 | 1 | 7 | 0 | 0 | 0 | 0 | 1 |
| GI.2 | 1 | 0 | 0 | 1 | 7 | 9 | 2 | 14 | 47 | 50 | 5 |
| GI.3 | 6 | 6 | 10 | 0 | 4 | 29 | 6 | 7 | 105 | 55 | 3 |
| GI.4 | 14 | 69 | 31 | 39 | 0 | 51 | 25 | 24 | 6 | 7 | 11 |
| GI.6 | 9 | 20 | 7 | 38 | 5 | 25 | 104 | 7 | 3 | 5 | 20 |
| GI.7 | 2 | 1 | 3 | 12 | 4 | 4 | 2 | 7 | 1 | 0 | 9 |
| GI.9 | 0 | 0 | 0 | 0 | 1 | 1 | 1 | 0 | 0 | 0 | 1 |
| GII.1 | 0 | 2 | 0 | 2 | 0 | 1 | 1 | 1 | 0 | 1 | 0 |
| GII.2 | 5 | 30 | 30 | 345 | 165 | 89 | 63 | 29 | 3 | 78 | 1333 |
| GII.3 | 12 | 83 | 37 | 67 | 531 | 29 | 20 | 63 | 283 | 295 | 41 |
| GII.4 | 1130 | 577 | 369 | 654 | 437 | 517 | 1097 | 641 | 528 | 825 | 307 |
| GII.5 | 0 | 1 | 0 | 0 | 0 | 9 | 0 | 0 | 2 | 1 | 2 |
| GII.6 | 11 | 3 | 141 | 19 | 4 | 27 | 27 | 337 | 8 | 43 | 113 |
| GII.7 | 0 | 1 | 0 | 10 | 5 | 4 | 19 | 5 | 1 | 15 | 9 |
| GII.11 | 0 | 0 | 0 | 0 | 0 | 0 | 0 | 0 | 0 | 0 | 0 |
| GII.12 | 0 | 0 | 8 | 31 | 45 | 44 | 4 | 0 | 2 | 0 | 0 |
| GII.13 | 0 | 0 | 0 | 14 | 0 | 3 | 3 | 12 | 30 | 6 | 1 |
| GII.14 | 50 | 40 | 1 | 26 | 66 | 97 | 51 | 56 | 7 | 0 | 0 |
| GII.17 | 0 | 0 | 1 | 0 | 0 | 0 | 2 | 2 | 220 | 309 | 105 |
| GII.21 | 0 | 0 | 0 | 0 | 0 | 0 | 0 | 1 | 0 | 0 | 0 |
| Total | 1241 | 837 | 639 | 1261 | 1275 | 946 | 1427 | 1206 | 1246 | 1690 | 1961 |

**Supplementary table S2:** Observed genotype proportions of HuNoV strains in Japan from season 2006/2007 to season 2016/2017 deposited in IASR.

| Genotype | 2006/2007 | 2007/2008 | 2008/2009 | 2009/2010 | 2010/2011 | 2011/2012 | 2012/2013 | 2013/2014 | 2014/2015 | 2015/2016 | 2016/2017 |
| --- | --- | --- | --- | --- | --- | --- | --- | --- | --- | --- | --- |
| GI.1 | 0.000806 | 0.004779 | 0.001565 | 0.002379 | 0.000784 | 0.007400 | 0 | 0 | 0 | 0 | 0.000510 |
| GI.2 | 0.000806 | 0 | 0 | 0.000793 | 0.005490 | 0.009514 | 0.001402 | 0.011609 | 0.037721 | 0.029586 | 0.002550 |
| GI.3 | 0.004835 | 0.007168 | 0.015649 | 0 | 0.003137 | 0.030655 | 0.004205 | 0.005804 | 0.084270 | 0.032544 | 0.001530 |
| GI.4 | 0.011281 | 0.082437 | 0.048513 | 0.030928 | 0 | 0.053911 | 0.017519 | 0.019900 | 0.004815 | 0.004142 | 0.005609 |
| GI.6 | 0.007252 | 0.023895 | 0.010955 | 0.030135 | 0.003922 | 0.026427 | 0.072880 | 0.005804 | 0.002408 | 0.002959 | 0.010199 |
| GI.7 | 0.001612 | 0.001195 | 0.004695 | 0.009516 | 0.003137 | 0.004228 | 0.001402 | 0.005804 | 0.000803 | 0 | 0.004589 |
| GI.9 | 0 | 0 | 0 | 0 | 0.000784 | 0.001057 | 0.000701 | 0 | 0 | 0 | 0.000510 |
| GII.1 | 0 | 0.002390 | 0 | 0.001586 | 0 | 0.001057 | 0.000701 | 0.000829 | 0 | 0.000592 | 0 |
| GII.2 | 0.004029 | 0.035842 | 0.046948 | 0.273592 | 0.129412 | 0.094080 | 0.044149 | 0.024046 | 0.002408 | 0.046154 | 0.679755 |
| GII.3 | 0.009670 | 0.099164 | 0.057903 | 0.053132 | 0.416471 | 0.030655 | 0.014015 | 0.052239 | 0.227127 | 0.174556 | 0.020908 |
| GII.4 | 0.910556 | 0.689367 | 0.577465 | 0.518636 | 0.342745 | 0.546512 | 0.768746 | 0.531509 | 0.423756 | 0.488166 | 0.156553 |
| GII.5 | 0 | 0.001195 | 0 | 0 | 0 | 0.009514 | 0 | 0 | 0.001605 | 0.000592 | 0.001020 |
| GII.6 | 0.008864 | 0.003584 | 0.220657 | 0.015067 | 0.003137 | 0.028541 | 0.018921 | 0.279436 | 0.006421 | 0.025444 | 0.057624 |
| GII.7 | 0 | 0.001195 | 0 | 0.007930 | 0.003922 | 0.004228 | 0.013315 | 0.004146 | 0.000803 | 0.008876 | 0.004589 |
| GII.11 | 0 | 0 | 0 | 0 | 0 | 0 | 0 | 0 | 0 | 0 | 0 |
| GII.12 | 0 | 0 | 0.012520 | 0.024584 | 0.035294 | 0.046512 | 0.002803 | 0 | 0.001605 | 0 | 0 |
| GII.13 | 0 | 0 | 0 | 0.011102 | 0 | 0.003171 | 0.002102 | 0.009950 | 0.024077 | 0.003550 | 0.000510 |
| GII.14 | 0.040290 | 0.047790 | 0.001565 | 0.020619 | 0.051765 | 0.102537 | 0.035739 | 0.046434 | 0.005618 | 0 | 0 |
| GII.17 | 0 | 0 | 0.001565 | 0 | 0 | 0 | 0.001402 | 0.001658 | 0.176565 | 0.182840 | 0.053544 |
| GII.21 | 0 | 0 | 0 | 0 | 0 | 0 | 0 | 0.000829 | 0 | 0 | 0 |
| Total | 1 | 1 | 1 | 1 | 1 | 1 | 1 | 1 | 1 | 1 | 1 |

**Supplementary table S3:** The INSD accession numbers of HuNoV sequences analyzed in the present study.

| Accession number | VP1 genotype^a^ | Isolation year | Isolation month | Isolation season |
| --- | --- | --- | --- | --- |
| KF039725 | GI.1 | 2010 | September | 2010/2011 |
| KF039726 | GI.1 | 2009 | October | 2009/2010 |
| KF039727 | GI-1 | 2009 | October | 2009/2010 |
| KF039728 | GI-1 | 2008 | May | 2007/2008 |
| KF039729 | GI-1 | 2010 | April | 2009/2010 |
| KF039730 | GI-1 | 2011 | April | 2010/2011 |
| KF039731 | GI-1 | 2009 | October | 2009/2010 |
| KF039732 | GI-1 | 2009 | October | 2009/2010 |
| KF039733 | GI-1 | 2011 | April | 2010/2011 |
| KF039734 | GI-1 | 2009 | October | 2009/2010 |
| KF039735 | GI-1 | 2008 | October | 2008/2009 |
| KF039736 | GI-1 | 2010 | September | 2010/2011 |
| KF039737 | GI-1 | 2009 | November | 2009/2010 |
| LC369137 | GI-1 | 2004 | October | 2004/2005 |
| KF306212 | GI-2 | 2013 | April | 2012/2013 |
| LC369138 | GI-2 | 2015 | March | 2014/2015 |
| JQ911594 | GI-3 | 2010 | March | 2009/2010 |
| KY934262 | GI-3 | 2015 | March | 2014/2015 |
| LC122714 | GI-3 | 2012 | February | 2011/2012 |
| LC369145 | GI-3 | 2015 | April | 2014/2015 |
| LC369151 | GI-3 | 2005 | December | 2005/2006 |
| LC122718 | GI-3 | 2011 | March | 2010/2011 |
| LC122703 | GI-4 | 2014 | June | 2013/2014 |
| LC369169 | GI-4 | 2008 | May | 2007/2008 |
| LC369172 | GI-4 | 2014 | January | 2013/2014 |
| LC369173 | GI-4 | 2014 | April | 2013/2014 |
| LC369174 | GI-4 | 2013 | December | 2013/2014 |
| LC122698 | GI-6 | 2013 | June | 2012/2013 |
| LC122699 | GI-6 | 2012 | June | 2011/2012 |
| LC122700 | GI-6 | 2012 | November | 2012/2013 |
| LC122691 | GI-6 | 2013 | April | 2012/2013 |
| LC369180 | GI-6 | 2008 | March | 2007/2008 |
| LC122692 | GI-6 | 2006 | March | 2005/2006 |
| KX907729 | GI-7 | 2011 | June | 2010/2011 |
| KX907730 | GI-7 | 2014 | February | 2013/2014 |
| KF586507 | GI-9 | 2012 | November | 2012/2013 |
| KX907731 | GI-9 | 2016 | January | 2015/2016 |
| LC369259 | GII-1 | 2001 | March | 2000/2001 |
| JX846925 | GII-2 | 1978 | May | 1977/1978 |
| KJ407074 | GII-2 | 2011 | January | 2010/2011 |
| KY771081 | GII-2 | 2016 | September | 2016/2017 |
| KY865306 | GII-2 | 2016 | November | 2016/2017 |
| KY865307 | GII-2 | 2016 | December | 2016/2017 |
| KY421121 | GII-2 | 2016 | December | 2016/2017 |
| KY421122 | GII-2 | 2016 | December | 2016/2017 |
| MF167650 | GII-2 | 2017 | March | 2016/2017 |
| MF167651 | GII-2 | 2017 | March | 2016/2017 |
| MF167652 | GII-2 | 2017 | March | 2016/2017 |
| LC209439 | GII-2 | 2014 | September | 2014/2015 |
| LC209463 | GII-2 | 2008 | March | 2007/2008 |
| LC209445 | GII-2 | 2012 | December | 2012/2013 |
| LC209449 | GII-2 | 2011 | July | 2010/2011 |
| LC209480 | GII-2 | 2010 | December | 2010/2011 |
| LC209481 | GII-2 | 2010 | December | 2010/2011 |
| LC209467 | GII-2 | 2011 | April | 2010/2011 |
| GU991355 | GII-3 | 2009 | January | 2008/2009 |
| KF306213 | GII-3 | 2013 | April | 2012/2013 |
| KF895841 | GII-3 | 2012 | March | 2011/2012 |
| KF944110 | GII-3 | 2011 | February | 2010/2011 |
| KF944111 | GII-3 | 2011 | February | 2010/2011 |
| KT779557 | GII-3 | 2012 | May | 2011/2012 |
| KY348697 | GII-3 | 2013 | November | 2013/2014 |
| KY348698 | GII-3 | 2014 | December | 2014/2015 |
| LC122740 | GII-3 | 2014 | February | 2013/2014 |
| LC122742 | GII-3 | 2010 | December | 2010/2011 |
| LC122760 | GII-3 | 2014 | December | 2014/2015 |
| LC122751 | GII-3 | 2004 | January | 2003/2004 |
| LC122752 | GII-3 | 2003 | December | 2003/2004 |
| LC122753 | GII-3 | 2004 | February | 2003/2004 |
| GU445325 | GII-4 | 2009 | November | 2009/2010 |
| GU991353 | GII-4 | 2008 | December | 2008/2009 |
| JN400599 | GII-4 | 2006 | January | 2005/2006 |
| JN400600 | GII-4 | 2006 | October | 2006/2007 |
| JN400601 | GII-4 | 2006 | October | 2006/2007 |
| JN400602 | GII-4 | 2006 | October | 2006/2007 |
| JN400603 | GII-4 | 2006 | October | 2006/2007 |
| JN400604 | GII-4 | 2006 | October | 2006/2007 |
| JN400605 | GII-4 | 2006 | October | 2006/2007 |
| JN400606 | GII-4 | 2006 | October | 2006/2007 |
| JN400607 | GII-4 | 2006 | November | 2006/2007 |
| JN400608 | GII-4 | 2006 | November | 2006/2007 |
| JN400609 | GII-4 | 2006 | November | 2006/2007 |
| JN400610 | GII-4 | 2007 | January | 2006/2007 |
| JN400611 | GII-4 | 2007 | January | 2006/2007 |
| JN400612 | GII-4 | 2007 | January | 2006/2007 |
| JN400613 | GII-4 | 2007 | April | 2006/2007 |
| JN400614 | GII-4 | 2007 | January | 2006/2007 |
| JN400615 | GII-4 | 2007 | January | 2006/2007 |
| JN400616 | GII-4 | 2008 | October | 2008/2009 |
| JN400617 | GII-4 | 2009 | May | 2008/2009 |
| JN400618 | GII-4 | 2009 | December | 2009/2010 |
| JN400619 | GII-4 | 2010 | January | 2009/2010 |
| JN400620 | GII-4 | 2010 | January | 2009/2010 |
| JN400621 | GII-4 | 2010 | January | 2009/2010 |
| JN400622 | GII-4 | 2010 | January | 2009/2010 |
| JN400623 | GII-4 | 2010 | January | 2009/2010 |
| JN400624 | GII-4 | 2010 | March | 2009/2010 |
| JN400625 | GII-4 | 2010 | March | 2009/2010 |
| JN400626 | GII-4 | 2010 | March | 2009/2010 |
| JN595867 | GII-4 | 2010 | May | 2009/2010 |
| JQ613552 | GII-4 | 2010 | August | 2009/2010 |
| JQ613572 | GII-4 | 2010 | April | 2009/2010 |
| JQ613573 | GII-4 | 2010 | November | 2010/2011 |
| JQ622197 | GII-4 | 2007 | February | 2006/2007 |
| JQ911595 | GII-4 | 2009 | March | 2008/2009 |
| JQ911596 | GII-4 | 2009 | April | 2008/2009 |
| JQ911597 | GII-4 | 2009 | July | 2008/2009 |
| JQ911598 | GII-4 | 2009 | May | 2008/2009 |
| JX439815 | GII-4 | 2010 | April | 2009/2010 |
| JX439816 | GII-4 | 2010 | May | 2009/2010 |
| JX439817 | GII-4 | 2010 | August | 2009/2010 |
| JX439818 | GII-4 | 2010 | November | 2010/2011 |
| JX439819 | GII-4 | 2011 | January | 2010/2011 |
| JX448566 | GII-4 | 2010 | May | 2009/2010 |
| JX459900 | GII-4 | 2011 | October | 2011/2012 |
| JX459901 | GII-4 | 2011 | October | 2011/2012 |
| JX459902 | GII-4 | 2012 | March | 2011/2012 |
| JX459903 | GII-4 | 2011 | February | 2010/2011 |
| JX459904 | GII-4 | 2011 | August | 2010/2011 |
| JX459905 | GII-4 | 2011 | August | 2010/2011 |
| JX459906 | GII-4 | 2011 | June | 2010/2011 |
| JX459907 | GII-4 | 2012 | May | 2011/2012 |
| JX459908 | GII-4 | 2012 | March | 2011/2012 |
| JX989073 | GII-4 | 2010 | November | 2010/2011 |
| JX989074 | GII-4 | 2011 | January | 2010/2011 |
| KC175323 | GII-4 | 2012 | August | 2011/2012 |
| KC175342 | GII-4 | 2009 | May | 2008/2009 |
| KC175343 | GII-4 | 2009 | May | 2008/2009 |
| KC175344 | GII-4 | 2009 | May | 2008/2009 |
| KC175345 | GII-4 | 2009 | May | 2008/2009 |
| KC175346 | GII-4 | 2009 | June | 2008/2009 |
| KC175347 | GII-4 | 2009 | June | 2008/2009 |
| KC175348 | GII-4 | 2009 | June | 2008/2009 |
| KC175349 | GII-4 | 2009 | June | 2008/2009 |
| KC175350 | GII-4 | 2009 | July | 2008/2009 |
| KC175351 | GII-4 | 2009 | July | 2008/2009 |
| KC175352 | GII-4 | 2009 | July | 2008/2009 |
| KC175353 | GII-4 | 2009 | July | 2008/2009 |
| KC175354 | GII-4 | 2009 | August | 2008/2009 |
| KC175355 | GII-4 | 2009 | August | 2008/2009 |
| KC175356 | GII-4 | 2009 | August | 2008/2009 |
| KC175357 | GII-4 | 2009 | August | 2008/2009 |
| KC175358 | GII-4 | 2009 | August | 2008/2009 |
| KC175359 | GII-4 | 2009 | August | 2008/2009 |
| KC175360 | GII-4 | 2009 | August | 2008/2009 |
| KC175361 | GII-4 | 2009 | August | 2008/2009 |
| KC175362 | GII-4 | 2009 | September | 2009/2010 |
| KC175363 | GII-4 | 2009 | September | 2009/2010 |
| KC175364 | GII-4 | 2009 | September | 2009/2010 |
| KC175365 | GII-4 | 2009 | September | 2009/2010 |
| KC175366 | GII-4 | 2009 | September | 2009/2010 |
| KC175367 | GII-4 | 2009 | September | 2009/2010 |
| KC175368 | GII-4 | 2009 | September | 2009/2010 |
| KC175369 | GII-4 | 2009 | October | 2009/2010 |
| KC175370 | GII-4 | 2009 | October | 2009/2010 |
| KC175371 | GII-4 | 2009 | October | 2009/2010 |
| KC175372 | GII-4 | 2009 | October | 2009/2010 |
| KC175373 | GII-4 | 2009 | November | 2009/2010 |
| KC175374 | GII-4 | 2009 | November | 2009/2010 |
| KC175375 | GII-4 | 2009 | November | 2009/2010 |
| KC175376 | GII-4 | 2009 | November | 2009/2010 |
| KC175377 | GII-4 | 2009 | November | 2009/2010 |
| KC175378 | GII-4 | 2009 | December | 2009/2010 |
| KC175379 | GII-4 | 2009 | December | 2009/2010 |
| KC175380 | GII-4 | 2010 | January | 2009/2010 |
| KC175381 | GII-4 | 2010 | January | 2009/2010 |
| KC175382 | GII-4 | 2010 | February | 2009/2010 |
| KC175383 | GII-4 | 2010 | February | 2009/2010 |
| KC175384 | GII-4 | 2010 | March | 2009/2010 |
| KC175385 | GII-4 | 2010 | March | 2009/2010 |
| KC175386 | GII-4 | 2010 | March | 2009/2010 |
| KC175387 | GII-4 | 2010 | March | 2009/2010 |
| KC175388 | GII-4 | 2009 | May | 2008/2009 |
| KC175389 | GII-4 | 2009 | May | 2008/2009 |
| KC175390 | GII-4 | 2009 | May | 2008/2009 |
| KC175391 | GII-4 | 2009 | May | 2008/2009 |
| KC175392 | GII-4 | 2009 | May | 2008/2009 |
| KC175393 | GII-4 | 2009 | May | 2008/2009 |
| KC175394 | GII-4 | 2009 | June | 2008/2009 |
| KC175395 | GII-4 | 2009 | June | 2008/2009 |
| KC175396 | GII-4 | 2009 | June | 2008/2009 |
| KC175397 | GII-4 | 2009 | July | 2008/2009 |
| KC175398 | GII-4 | 2009 | July | 2008/2009 |
| KC175399 | GII-4 | 2009 | July | 2008/2009 |
| KC175400 | GII-4 | 2009 | July | 2008/2009 |
| KC175401 | GII-4 | 2009 | August | 2008/2009 |
| KC175402 | GII-4 | 2009 | August | 2008/2009 |
| KC175403 | GII-4 | 2009 | August | 2008/2009 |
| KC175404 | GII-4 | 2009 | August | 2008/2009 |
| KC175405 | GII-4 | 2009 | August | 2008/2009 |
| KC175406 | GII-4 | 2009 | September | 2009/2010 |
| KC175407 | GII-4 | 2009 | September | 2009/2010 |
| KC175408 | GII-4 | 2009 | September | 2009/2010 |
| KC175409 | GII-4 | 2009 | September | 2009/2010 |
| KC175410 | GII-4 | 2009 | September | 2009/2010 |
| KC409238 | GII-4 | 2009 | June | 2008/2009 |
| KC409239 | GII-4 | 2009 | July | 2008/2009 |
| KC409240 | GII-4 | 2010 | March | 2009/2010 |
| KC409241 | GII-4 | 2010 | April | 2009/2010 |
| KC409242 | GII-4 | 2010 | April | 2009/2010 |
| KC409243 | GII-4 | 2010 | April | 2009/2010 |
| KC409244 | GII-4 | 2010 | April | 2009/2010 |
| KC409245 | GII-4 | 2009 | June | 2008/2009 |
| KC409246 | GII-4 | 2009 | July | 2008/2009 |
| KC409247 | GII-4 | 2009 | July | 2008/2009 |
| KC409248 | GII-4 | 2009 | August | 2008/2009 |
| KC409249 | GII-4 | 2009 | August | 2008/2009 |
| KC409250 | GII-4 | 2009 | September | 2009/2010 |
| KC409251 | GII-4 | 2009 | September | 2009/2010 |
| KC409252 | GII-4 | 2009 | September | 2009/2010 |
| KC409253 | GII-4 | 2009 | September | 2009/2010 |
| KC409254 | GII-4 | 2009 | September | 2009/2010 |
| KC409255 | GII-4 | 2009 | September | 2009/2010 |
| KC409256 | GII-4 | 2009 | September | 2009/2010 |
| KC409257 | GII-4 | 2009 | September | 2009/2010 |
| KC409258 | GII-4 | 2009 | September | 2009/2010 |
| KC409259 | GII-4 | 2009 | September | 2009/2010 |
| KC409260 | GII-4 | 2009 | September | 2009/2010 |
| KC409261 | GII-4 | 2009 | September | 2009/2010 |
| KC409262 | GII-4 | 2009 | September | 2009/2010 |
| KC409263 | GII-4 | 2009 | September | 2009/2010 |
| KC409264 | GII-4 | 2009 | September | 2009/2010 |
| KC409265 | GII-4 | 2009 | September | 2009/2010 |
| KC409266 | GII-4 | 2009 | October | 2009/2010 |
| KC409267 | GII-4 | 2009 | October | 2009/2010 |
| KC409268 | GII-4 | 2009 | October | 2009/2010 |
| KC409269 | GII-4 | 2009 | October | 2009/2010 |
| KC409270 | GII-4 | 2009 | October | 2009/2010 |
| KC409271 | GII-4 | 2009 | October | 2009/2010 |
| KC409272 | GII-4 | 2009 | October | 2009/2010 |
| KC409273 | GII-4 | 2009 | October | 2009/2010 |
| KC409274 | GII-4 | 2009 | October | 2009/2010 |
| KC409275 | GII-4 | 2009 | October | 2009/2010 |
| KC409276 | GII-4 | 2009 | October | 2009/2010 |
| KC409277 | GII-4 | 2009 | October | 2009/2010 |
| KC409278 | GII-4 | 2009 | October | 2009/2010 |
| KC409279 | GII-4 | 2009 | October | 2009/2010 |
| KC409280 | GII-4 | 2009 | October | 2009/2010 |
| KC409281 | GII-4 | 2009 | October | 2009/2010 |
| KC409282 | GII-4 | 2009 | October | 2009/2010 |
| KC409283 | GII-4 | 2009 | November | 2009/2010 |
| KC409284 | GII-4 | 2009 | November | 2009/2010 |
| KC409285 | GII-4 | 2009 | November | 2009/2010 |
| KC409286 | GII-4 | 2009 | November | 2009/2010 |
| KC409287 | GII-4 | 2009 | November | 2009/2010 |
| KC409288 | GII-4 | 2009 | November | 2009/2010 |
| KC409289 | GII-4 | 2009 | November | 2009/2010 |
| KC409290 | GII-4 | 2009 | December | 2009/2010 |
| KC409291 | GII-4 | 2009 | December | 2009/2010 |
| KC409292 | GII-4 | 2009 | December | 2009/2010 |
| KC409293 | GII-4 | 2010 | January | 2009/2010 |
| KC409294 | GII-4 | 2010 | January | 2009/2010 |
| KC409295 | GII-4 | 2010 | January | 2009/2010 |
| KC409296 | GII-4 | 2010 | January | 2009/2010 |
| KC409297 | GII-4 | 2010 | February | 2009/2010 |
| KC409298 | GII-4 | 2010 | February | 2009/2010 |
| KC409299 | GII-4 | 2010 | March | 2009/2010 |
| KC409300 | GII-4 | 2010 | March | 2009/2010 |
| KC409301 | GII-4 | 2010 | March | 2009/2010 |
| KC409302 | GII-4 | 2010 | March | 2009/2010 |
| KC409303 | GII-4 | 2010 | March | 2009/2010 |
| KC409304 | GII-4 | 2009 | May | 2008/2009 |
| KC409305 | GII-4 | 2009 | June | 2008/2009 |
| KC409306 | GII-4 | 2009 | June | 2008/2009 |
| KC409307 | GII-4 | 2009 | June | 2008/2009 |
| KC409308 | GII-4 | 2009 | July | 2008/2009 |
| KC409309 | GII-4 | 2009 | July | 2008/2009 |
| KC409310 | GII-4 | 2009 | July | 2008/2009 |
| KC409311 | GII-4 | 2009 | September | 2009/2010 |
| KC409312 | GII-4 | 2009 | September | 2009/2010 |
| KC409313 | GII-4 | 2009 | October | 2009/2010 |
| KC409314 | GII-4 | 2009 | October | 2009/2010 |
| KC409315 | GII-4 | 2009 | October | 2009/2010 |
| KC409316 | GII-4 | 2009 | October | 2009/2010 |
| KC409317 | GII-4 | 2009 | October | 2009/2010 |
| KC409318 | GII-4 | 2009 | November | 2009/2010 |
| KC517361 | GII-4 | 2012 | January | 2011/2012 |
| KC517362 | GII-4 | 2012 | January | 2011/2012 |
| KC517364 | GII-4 | 2012 | January | 2011/2012 |
| KC517365 | GII-4 | 2012 | February | 2011/2012 |
| KC517368 | GII-4 | 2012 | April | 2011/2012 |
| KC517369 | GII-4 | 2012 | April | 2011/2012 |
| KC517372 | GII-4 | 2012 | July | 2011/2012 |
| KC517376 | GII-4 | 2012 | August | 2011/2012 |
| KC517377 | GII-4 | 2012 | September | 2012/2013 |
| KC517378 | GII-4 | 2012 | September | 2012/2013 |
| KC576909 | GII-4 | 2011 | March | 2010/2011 |
| KC576912 | GII-4 | 2011 | January | 2010/2011 |
| KC577174 | GII-4 | 2011 | October | 2011/2012 |
| KC631827 | GII-4 | 2012 | December | 2012/2013 |
| KC894942 | GII-4 | 2011 | January | 2010/2011 |
| KC894943 | GII-4 | 2011 | January | 2010/2011 |
| KC960615 | GII-4 | 2009 | October | 2009/2010 |
| KC962453 | GII-4 | 2010 | July | 2009/2010 |
| KC962462 | GII-4 | 2011 | November | 2011/2012 |
| KF306214 | GII-4 | 2013 | April | 2012/2013 |
| KF429760 | GII-4 | 2012 | July | 2011/2012 |
| KF429766 | GII-4 | 2011 | December | 2011/2012 |
| KF429768 | GII-4 | 2012 | October | 2012/2013 |
| KF429776 | GII-4 | 2012 | February | 2011/2012 |
| KF429777 | GII-4 | 2012 | March | 2011/2012 |
| KF429778 | GII-4 | 2012 | February | 2011/2012 |
| KF429787 | GII-4 | 2012 | August | 2011/2012 |
| KF429790 | GII-4 | 2012 | May | 2011/2012 |
| KF712497 | GII-4 | 2012 | April | 2011/2012 |
| KF712499 | GII-4 | 2012 | October | 2012/2013 |
| KF712501 | GII-4 | 2012 | September | 2012/2013 |
| KJ407073 | GII-4 | 2012 | May | 2011/2012 |
| KJ407075 | GII-4 | 2012 | March | 2011/2012 |
| KJ541743 | GII-4 | 2011 | April | 2010/2011 |
| KJ649705 | GII-4 | 2013 | February | 2012/2013 |
| KJ685403 | GII-4 | 2011 | July | 2010/2011 |
| KJ685405 | GII-4 | 2011 | August | 2010/2011 |
| KJ685408 | GII-4 | 2011 | March | 2010/2011 |
| KJ685412 | GII-4 | 2012 | January | 2011/2012 |
| KJ685413 | GII-4 | 2011 | November | 2011/2012 |
| KJ685414 | GII-4 | 2010 | August | 2009/2010 |
| KJ685415 | GII-4 | 2011 | July | 2010/2011 |
| KJ685417 | GII-4 | 2011 | November | 2011/2012 |
| KJ710245 | GII-4 | 2011 | January | 2010/2011 |
| KJ955492 | GII-4 | 2012 | December | 2012/2013 |
| KJ955493 | GII-4 | 2012 | December | 2012/2013 |
| KM258128 | GII-4 | 2012 | March | 2011/2012 |
| KM258129 | GII-4 | 2012 | September | 2012/2013 |
| KM258130 | GII-4 | 2012 | March | 2011/2012 |
| KM258131 | GII-4 | 2012 | March | 2011/2012 |
| KM272334 | GII-4 | 2012 | August | 2011/2012 |
| KT202793 | GII-4 | 2013 | November | 2013/2014 |
| KT202794 | GII-4 | 2014 | February | 2013/2014 |
| KT202795 | GII-4 | 2014 | March | 2013/2014 |
| KT202796 | GII-4 | 2014 | March | 2013/2014 |
| KT202797 | GII-4 | 2014 | November | 2014/2015 |
| KT202798 | GII-4 | 2014 | November | 2014/2015 |
| KX907727 | GII-4 | 2015 | November | 2015/2016 |
| KY496327 | GII-4 | 2012 | November | 2012/2013 |
| KY947546 | GII-4 | 2015 | January | 2014/2015 |
| KY947547 | GII-4 | 2014 | November | 2014/2015 |
| KY947549 | GII-4 | 2016 | March | 2015/2016 |
| KY947550 | GII-4 | 2015 | November | 2015/2016 |
| KX586330 | GII-4 | 2015 | July | 2014/2015 |
| LC122773 | GII-4 | 2008 | March | 2007/2008 |
| LC369297 | GII-4 | 2015 | April | 2014/2015 |
| LC122816 | GII-4 | 2013 | December | 2013/2014 |
| LC369330 | GII-4 | 2014 | December | 2014/2015 |
| LC122823 | GII-4 | 2012 | December | 2012/2013 |
| LC122829 | GII-4 | 2011 | December | 2011/2012 |
| LC122801 | GII-4 | 2012 | December | 2012/2013 |
| LC369345 | GII-4 | 2015 | January | 2014/2015 |
| LC369352 | GII-4 | 2014 | February | 2013/2014 |
| LC369356 | GII-4 | 2015 | October | 2015/2016 |
| LC369362 | GII-4 | 2014 | November | 2014/2015 |
| LC369365 | GII-4 | 2014 | December | 2014/2015 |
| LC369369 | GII-4 | 2014 | December | 2014/2015 |
| LC122859 | GII-5 | 2006 | March | 2005/2006 |
| JX989075 | GII-6 | 2011 | January | 2010/2011 |
| KJ407072 | GII-6 | 2010 | December | 2010/2011 |
| KU935739 | GII-6 | 2015 | September | 2015/2016 |
| LC122895 | GII-6 | 2008 | December | 2008/2009 |
| LC122923 | GII-6 | 2004 | November | 2004/2005 |
| LC122910 | GII-6 | 2014 | April | 2013/2014 |
| LC122906 | GII-6 | 2014 | June | 2013/2014 |
| LC122920 | GII-6 | 2004 | November | 2004/2005 |
| LC369400 | GII-6 | 2014 | April | 2013/2014 |
| LC369403 | GII-6 | 2014 | May | 2013/2014 |
| LC369411 | GII-6 | 2014 | March | 2013/2014 |
| LC369421 | GII-6 | 2014 | May | 2013/2014 |
| LC369423 | GII-6 | 2013 | April | 2012/2013 |
| LC122886 | GII-7 | 2012 | February | 2011/2012 |
| HQ392821 | GII-11 | 2009 | August | 2008/2009 |
| JQ613568 | GII-12 | 2009 | May | 2008/2009 |
| KC464496 | GII-12 | 2010 | January | 2009/2010 |
| KC464497 | GII-12 | 2010 | January | 2009/2010 |
| KC464499 | GII-12 | 2010 | January | 2009/2010 |
| KC464500 | GII-12 | 2010 | January | 2009/2010 |
| LC369194 | GII-12 | 2005 | February | 2004/2005 |
| KY947548 | GII-13 | 2016 | January | 2015/2016 |
| LC122860 | GII-14 | 2012 | February | 2011/2012 |
| LC122875 | GII-14 | 2010 | May | 2009/2010 |
| LC122874 | GII-14 | 2012 | April | 2011/2012 |
| LC122885 | GII-14 | 2008 | February | 2007/2008 |
| LC122877 | GII-14 | 2011 | May | 2010/2011 |
| LC122869 | GII-14 | 2013 | November | 2013/2014 |
| LC122870 | GII-14 | 2013 | November | 2013/2014 |
| LC122871 | GII-14 | 2012 | June | 2011/2012 |
| LC122861 | GII-14 | 2012 | June | 2011/2012 |
| LC122862 | GII-14 | 2012 | November | 2012/2013 |
| LC369212 | GII-14 | 2012 | February | 2011/2012 |
| KP998539 | GII-17 | 2014 | December | 2014/2015 |
| KT970370 | GII-17 | 2014 | December | 2014/2015 |
| KT970372 | GII-17 | 2015 | January | 2014/2015 |
| KT970373 | GII-17 | 2015 | January | 2014/2015 |
| KT970374 | GII-17 | 2015 | January | 2014/2015 |
| KT970376 | GII-17 | 2015 | January | 2014/2015 |
| KT970377 | GII-17 | 2015 | March | 2014/2015 |
| KT992785 | GII-17 | 2015 | March | 2014/2015 |
| KT992787 | GII-17 | 2015 | March | 2014/2015 |
| KT992788 | GII-17 | 2015 | March | 2014/2015 |
| KT992790 | GII-17 | 2015 | March | 2014/2015 |
| LC369214 | GII-17 | 2014 | December | 2014/2015 |
| LC369219 | GII-17 | 2015 | February | 2014/2015 |
| LC369236 | GII-17 | 2015 | January | 2014/2015 |
| LC369244 | GII-17 | 2015 | May | 2014/2015 |
| LC369254 | GII-17 | 2015 | February | 2014/2015 |
| KX079488 | GII-21 | 2015 | April | 2014/2015 |
| LC369262 | GII-21 | 2006 | February | 2005/2006 |

^a^The VP1 genotype was determined by the Norovirus Genotyping Tool (version 1.0) (Kroneman et al., 2011).

**Supplementary table S4:** Numbers of HuNoV sequences analyzed in the present study classified according to the isolation season and the VP1 genotype.

| Genotype^a^ | Before^b^ | 2006/2007 | 2007/2008 | 2008/2009 | 2009/2010 | 2010/2011 | 2011/2012 | 2012/2013 | 2013/2014 | 2014/2015 | 2015/2016 | 2016/2017 |
| --- | --- | --- | --- | --- | --- | --- | --- | --- | --- | --- | --- | --- |
| GI.1 | 1 (2004/2005) | 0 | 1 | 1 | 7 | 4 | 0 | 0 | 0 | 0 | 0 | 0 |
| GI.2 |  | 0 | 0 | 0 | 0 | 0 | 0 | 1 | 0 | 1 | 0 | 0 |
| GI.3 | 1 (2005/2006) | 0 | 0 | 0 | 1 | 1 | 1 | 0 | 0 | 2 | 0 | 0 |
| GI.4 |  | 0 | 1 | 0 | 0 | 0 | 0 | 0 | 4 | 0 | 0 | 0 |
| GI.6 | 1 (2005/2006) | 0 | 1 | 0 | 0 | 0 | 1 | 3 | 0 | 0 | 0 | 0 |
| GI.7 |  | 0 | 0 | 0 | 0 | 1 | 0 | 0 | 1 | 0 | 0 | 0 |
| GI.9 |  | 0 | 0 | 0 | 0 | 0 | 0 | 1 | 0 | 0 | 1 | 0 |
| GII.1 | 1 (2000/2001) | 0 | 0 | 0 | 0 | 0 | 0 | 0 | 0 | 0 | 0 | 0 |
| GII.2 | 1 (1977/1978) | 0 | 1 | 0 | 0 | 5 | 0 | 1 | 0 | 1 | 0 | 8 |
| GII.3 | 3 (2003/2004) | 0 | 0 | 1 | 0 | 3 | 2 | 1 | 2 | 2 | 0 | 0 |
| GII.4 |  | 17 | 1 | 59 | 118 | 19 | 34 | 14 | 6 | 11 | 4 | 0 |
| GII.5 | 1 (2005/2006) | 0 | 0 | 0 | 0 | 0 | 0 | 0 | 0 | 0 | 0 | 0 |
| GII.6 | 2 (2004/2005) | 0 | 0 | 1 | 0 | 2 | 0 | 1 | 6 | 0 | 1 | 0 |
| GII.7 |  | 0 | 0 | 0 | 0 | 0 | 1 | 0 | 0 | 0 | 0 | 0 |
| GII.11 |  | 0 | 0 | 1 | 0 | 0 | 0 | 0 | 0 | 0 | 0 | 0 |
| GII.12 | 1 (2004/2005) | 0 | 0 | 1 | 4 | 0 | 0 | 0 | 0 | 0 | 0 | 0 |
| GII.13 |  | 0 | 0 | 0 | 0 | 0 | 0 | 0 | 0 | 0 | 1 | 0 |
| GII.14 |  | 0 | 1 | 0 | 1 | 1 | 5 | 1 | 2 | 0 | 0 | 0 |
| GII.17 |  | 0 | 0 | 0 | 0 | 0 | 0 | 0 | 0 | 16 | 0 | 0 |
| GII.21 | 1 (2005/2006) | 0 | 0 | 0 | 0 | 0 | 0 | 0 | 0 | 1 | 0 | 0 |
| Total | 13 | 17 | 6 | 64 | 131 | 36 | 44 | 23 | 21 | 34 | 7 | 8 |

^a^The VP1 genotype was determined by the Norovirus Genotyping Tool (version 1.0) (Kroneman et al., 2011).

^b^Numbers of sequences available in the most recent season (indicated in the parentheses) before season 2006/2007.

**Supplementary table S5:** Compensation for unavailable sequence data within each genotype.

| Genotype^a^ | Before^b^ | 2006/2007 | 2007/2008 | 2008/2009 | 2009/2010 | 2010/2011 | 2011/2012 | 2012/2013 | 2013/2014 | 2014/2015 | 2015/2016 | 2016/2017 |
| --- | --- | --- | --- | --- | --- | --- | --- | --- | --- | --- | --- | --- |
| GI.1^c^ | 1 (2004/2005) |  | 1 | 1 | 7 | 4 |  |  |  |  |  |  |
| GI.2 |  |  |  |  |  |  |  | 1 |  | 1 |  |  |
| GI.3 | 1 (2005/2006) |  |  |  | 1 | 1 | 1 |  |  | 2 |  |  |
| GI.4 |  |  | 1 |  |  |  |  |  | 4 |  |  |  |
| GI.6 | 1 (2005/2006) |  | 1 |  |  |  | 1 | 3 |  |  |  |  |
| GI.7 |  |  |  |  |  | 1 |  |  | 1 |  |  |  |
| GI.9 |  |  |  |  |  |  |  | 1 |  |  | 1 |  |
| GII.1 | 1 (2000/2001) |  |  |  |  |  |  |  |  |  |  |  |
| GII.2 | 1 (1977/1978) |  | 1 |  |  | 5 |  | 1 |  | 1 |  | 8 |
| GII.3 | 3 (2003/2004) |  |  | 1 |  | 3 | 2 | 1 | 2 | 2 |  |  |
| GII.4 |  | 17 | 1 | 59 | 118 | 19 | 34 | 14 | 6 | 11 | 4 |  |
| GII.5 | 1 (2005/2006) |  |  |  |  |  |  |  |  |  |  |  |
| GII.6 | 2 (2004/2005) |  |  | 1 |  | 2 |  | 1 | 6 |  | 1 |  |
| GII.7 |  |  |  |  |  |  | 1 |  |  |  |  |  |
| GII.11 |  |  |  | 1 |  |  |  |  |  |  |  |  |
| GII.12 | 1 (2004/2005) |  |  | 1 | 4 |  |  |  |  |  |  |  |
| GII.13 |  |  |  |  |  |  |  |  |  |  | 1 |  |
| GII.14 |  |  | 1 |  | 1 | 1 | 5 | 1 | 2 |  |  |  |
| GII.17 |  |  |  |  |  |  |  |  |  | 16 |  |  |
| GII.21 | 1 (2005/2006) |  |  |  |  |  |  |  |  | 1 |  |  |
| Total | 13 | 17 | 6 | 64 | 131 | 36 | 44 | 23 | 21 | 34 | 7 | 8 |

^a^The VP1 genotype was determined by the Norovirus Genotyping Tool (version 1.0) (Kroneman et al., 2011).

^b^Numbers of sequences available in the most recent season (indicated in the parentheses) before season 2006/2007.

^c^Unavailable sequence data in a particular season were compensated by the sequence data available in the closest season before (→) or after (←) that season or both (⇄) within each genotype.

**Supplementary table S6:** Predicted genotype proportions in season 2008/2009.

| Genotype | Pre-target | Target | VP1 | | | | VP1, VP2, and NS | | | |
| --- | --- | --- | --- | --- | --- | --- | --- | --- | --- | --- |
|  | 2007/2008 | 2008/2009 | 0 | 0.000005 | 0.00005 | 0.0005 | 0 | 0.000005 | 0.00005 | 0.0005 |
| GI.1 | 0.004779^a^ | 0.001565^b^ | 0.046764^c^ | 0.046724 | 0.046425 | 0.043287 | 0.050714 | 0.051416 | 0.050957 | 0.047399 |
| GI.2 | 0 | 0 | 0 | 0.000055 | 0.000549 | 0.005046 | 0 | 0.000064 | 0.000628 | 0.005726 |
| GI.3 | 0.007168 | 0.015649 | 0.069337 | 0.069278 | 0.068838 | 0.064220 | 0.074918 | 0.075932 | 0.075263 | 0.070059 |
| GI.4 | 0.082437 | 0.048513 | 0.072880 | 0.072918 | 0.073076 | 0.075937 | 0.036933 | 0.035146 | 0.035747 | 0.038249 |
| GI.6 | 0.023895 | 0.010955 | 0.117904 | 0.117849 | 0.117383 | 0.112887 | 0.103068 | 0.102709 | 0.102412 | 0.099293 |
| GI.7 | 0.001195 | 0.004695 | 0.012575 | 0.012563 | 0.012480 | 0.011598 | 0.013955 | 0.014175 | 0.014038 | 0.013003 |
| GI.9 | 0 | 0 | 0 | 0.000056 | 0.000558 | 0.005079 | 0 | 0.000065 | 0.000642 | 0.005775 |
| GII.1 | 0.002390 | 0 | 0.025216 | 0.025190 | 0.024997 | 0.023008 | 0.028007 | 0.028447 | 0.028132 | 0.025723 |
| GII.2 | 0.035842 | 0.046948 | 0.142507 | 0.142457 | 0.142002 | 0.137883 | 0.116218 | 0.115063 | 0.115148 | 0.112869 |
| GII.3 | 0.099164 | 0.057903 | 0.392043 | 0.391893 | 0.390541 | 0.378287 | 0.474117 | 0.474814 | 0.472268 | 0.454990 |
| GII.4 | 0.689367 | 0.577465 | 0.000000 | 0.000000 | 0.000000 | 0.000000 | 0.000000 | 0.000000 | 0.000000 | 0.000000 |
| GII.5 | 0.001195 | 0 | 0.013034 | 0.013021 | 0.012919 | 0.011874 | 0.014630 | 0.014873 | 0.014703 | 0.013418 |
| GII.6 | 0.003584 | 0.220657 | 0.036612 | 0.036580 | 0.036341 | 0.033820 | 0.040248 | 0.040852 | 0.040468 | 0.037551 |
| GII.7 | 0.001195 | 0 | 0.013034 | 0.013021 | 0.012919 | 0.011874 | 0.014630 | 0.014873 | 0.014703 | 0.013418 |
| GII.11 | 0 | 0 | 0 | 0.000056 | 0.000558 | 0.005079 | 0 | 0.000065 | 0.000642 | 0.005775 |
| GII.12 | 0 | 0.012520 | 0 | 0.000056 | 0.000559 | 0.005140 | 0 | 0.000065 | 0.000643 | 0.005869 |
| GII.13 | 0 | 0 | 0 | 0.000056 | 0.000558 | 0.005079 | 0 | 0.000065 | 0.000642 | 0.005775 |
| GII.14 | 0.047790 | 0.001565 | 0.058094 | 0.058114 | 0.058178 | 0.059628 | 0.032562 | 0.031247 | 0.031676 | 0.033295 |
| GII.17 | 0 | 0.001565 | 0 | 0.000056 | 0.000559 | 0.005135 | 0 | 0.000065 | 0.000644 | 0.005943 |
| GII.21 | 0 | 0 | 0 | 0.000056 | 0.000559 | 0.005140 | 0 | 0.000065 | 0.000643 | 0.005869 |
| Total | 1 | 1 | 1 | 1 | 1 | 1 | 1 | 1 | 1 | 1 |

^a^Observed genotype proportions in the pre-target season.

^b^Observed genotype proportions in the target season.

^c^Cells are colored green or blue when the predicted directions of changes in genotype proportions are correct or incorrect, respectively.

**Supplementary table S7:** Predicted genotype proportions in season 2009/2010.

| Genotype | Pre-target | Target | VP1 | | | | VP1, VP2, and NS | | | |
| --- | --- | --- | --- | --- | --- | --- | --- | --- | --- | --- |
|  | 2008/2009 | 2009/2010 | 0 | 0.000005 | 0.00005 | 0.0005 | 0 | 0.000005 | 0.00005 | 0.0005 |
| GI.1 | 0.001565^a^ | 0.002379^b^ | 0.002332^c^ | 0.002331 | 0.002337 | 0.002318 | 0.002290 | 0.002676 | 0.002549 | 0.002503 |
| GI.2 | 0 | 0.000793 | 0 | 0.000007 | 0.000075 | 0.000744 | 0 | 0.000008 | 0.000081 | 0.000801 |
| GI.3 | 0.015649 | 0 | 0.023294 | 0.023286 | 0.023347 | 0.023152 | 0.022838 | 0.027305 | 0.025542 | 0.025031 |
| GI.4 | 0.048513 | 0.030928 | 0.069496 | 0.069469 | 0.069656 | 0.069025 | 0.067647 | 0.079845 | 0.075785 | 0.073958 |
| GI.6 | 0.010955 | 0.030135 | 0.016186 | 0.016181 | 0.016222 | 0.016089 | 0.015915 | 0.018684 | 0.017710 | 0.017378 |
| GI.7 | 0.004695 | 0.009516 | 0.006991 | 0.006989 | 0.007007 | 0.006950 | 0.006871 | 0.008030 | 0.007691 | 0.007526 |
| GI.9 | 0 | 0 | 0 | 0.000008 | 0.000075 | 0.000748 | 0 | 0.000009 | 0.000082 | 0.000807 |
| GII.1 | 0 | 0.001586 | 0 | 0.000007 | 0.000068 | 0.000687 | 0 | 0.000007 | 0.000055 | 0.000583 |
| GII.2 | 0.046948 | 0.273592 | 0.063834 | 0.063847 | 0.063820 | 0.063701 | 0.066232 | 0.060880 | 0.057109 | 0.062890 |
| GII.3 | 0.057903 | 0.053132 | 0.075932 | 0.075951 | 0.075888 | 0.075799 | 0.073861 | 0.072967 | 0.060135 | 0.062263 |
| GII.4 | 0.577465 | 0.518636 | 0.435791 | 0.435710 | 0.435055 | 0.432081 | 0.432460 | 0.437383 | 0.445485 | 0.421487 |
| GII.5 | 0 | 0 | 0 | 0.000007 | 0.000069 | 0.000693 | 0 | 0.000006 | 0.000071 | 0.000762 |
| GII.6 | 0.220657 | 0.015067 | 0.284842 | 0.284870 | 0.284814 | 0.283941 | 0.290143 | 0.271834 | 0.289395 | 0.301920 |
| GII.7 | 0 | 0.007930 | 0 | 0.000007 | 0.000069 | 0.000688 | 0 | 0.000007 | 0.000069 | 0.000724 |
| GII.11 | 0 | 0 | 0 | 0.000007 | 0.000070 | 0.000699 | 0 | 0.000007 | 0.000075 | 0.000782 |
| GII.12 | 0.012520 | 0.024584 | 0.017030 | 0.017036 | 0.017017 | 0.017010 | 0.017296 | 0.016255 | 0.013942 | 0.014896 |
| GII.13 | 0 | 0.011102 | 0 | 0.000007 | 0.000070 | 0.000702 | 0 | 0.000007 | 0.000062 | 0.000680 |
| GII.14 | 0.001565 | 0.020619 | 0.002123 | 0.002124 | 0.002123 | 0.002120 | 0.002183 | 0.002088 | 0.002138 | 0.002229 |
| GII.17 | 0.001565 | 0 | 0.002148 | 0.002149 | 0.002147 | 0.002146 | 0.002264 | 0.001997 | 0.001966 | 0.002170 |
| GII.21 | 0 | 0 | 0 | 0.000007 | 0.000071 | 0.000706 | 0 | 0.000007 | 0.000056 | 0.000610 |
| Total | 1 | 1 | 1 | 1 | 1 | 1 | 1 | 1 | 1 | 1 |

^a^Observed genotype proportions in the pre-target season.

^b^Observed genotype proportions in the target season.

^c^Cells are colored green or blue when the predicted directions of changes in genotype proportions are correct or incorrect, respectively.

**Supplementary table S8:** Predicted genotype proportions in season 2010/2011.

| Genotype | Pre-target | Target | VP1 | | | | VP1, VP2, and NS | | | |
| --- | --- | --- | --- | --- | --- | --- | --- | --- | --- | --- |
|  | 2009/2010 | 2010/2011 | 0 | 0.000005 | 0.00005 | 0.0005 | 0 | 0.000005 | 0.00005 | 0.0005 |
| GI.1 | 0.002379^a^ | 0.000784^b^ | 0.002655^c^ | 0.002655 | 0.002656 | 0.002655 | 0.002613 | 0.002654 | 0.002649 | 0.002621 |
| GI.2 | 0.000793 | 0.005490 | 0.000885 | 0.000885 | 0.000886 | 0.000885 | 0.000872 | 0.000885 | 0.000884 | 0.000874 |
| GI.3 | 0 | 0.003137 | 0 | 0.000006 | 0.000055 | 0.000556 | 0 | 0.000006 | 0.000055 | 0.000549 |
| GI.4 | 0.030928 | 0 | 0.033404 | 0.033404 | 0.033404 | 0.033346 | 0.032951 | 0.033423 | 0.033179 | 0.032959 |
| GI.6 | 0.030135 | 0.003922 | 0.032944 | 0.032944 | 0.032960 | 0.032953 | 0.032470 | 0.032894 | 0.032928 | 0.032566 |
| GI.7 | 0.009516 | 0.003137 | 0.010514 | 0.010514 | 0.010519 | 0.010517 | 0.010357 | 0.010504 | 0.010504 | 0.010389 |
| GI.9 | 0 | 0.000784 | 0 | 0.000006 | 0.000056 | 0.000560 | 0 | 0.000006 | 0.000056 | 0.000553 |
| GII.1 | 0.001586 | 0 | 0.001768 | 0.001768 | 0.001769 | 0.001767 | 0.001741 | 0.001767 | 0.001765 | 0.001746 |
| GII.2 | 0.273592 | 0.129412 | 0.285434 | 0.285433 | 0.285568 | 0.285471 | 0.282539 | 0.284442 | 0.285839 | 0.283008 |
| GII.3 | 0.053132 | 0.416471 | 0.057795 | 0.057794 | 0.057822 | 0.057811 | 0.056973 | 0.057692 | 0.057766 | 0.057133 |
| GII.4 | 0.518636 | 0.342745 | 0.487520 | 0.487489 | 0.486968 | 0.484172 | 0.493693 | 0.488721 | 0.487219 | 0.489379 |
| GII.5 | 0 | 0 | 0 | 0.000006 | 0.000056 | 0.000560 | 0 | 0.000006 | 0.000056 | 0.000553 |
| GII.6 | 0.015067 | 0.003137 | 0.016053 | 0.016053 | 0.016060 | 0.016052 | 0.015845 | 0.016016 | 0.016033 | 0.015874 |
| GII.7 | 0.007930 | 0.003922 | 0.008803 | 0.008803 | 0.008806 | 0.008800 | 0.008669 | 0.008796 | 0.008792 | 0.008692 |
| GII.11 | 0 | 0 | 0 | 0.000006 | 0.000056 | 0.000560 | 0 | 0.000006 | 0.000056 | 0.000553 |
| GII.12 | 0.024584 | 0.035294 | 0.027255 | 0.027254 | 0.027265 | 0.027250 | 0.026833 | 0.027241 | 0.027186 | 0.026903 |
| GII.13 | 0.011102 | 0 | 0.012302 | 0.012302 | 0.012306 | 0.012295 | 0.012115 | 0.012291 | 0.012287 | 0.012145 |
| GII.14 | 0.020619 | 0.051765 | 0.022668 | 0.022668 | 0.022678 | 0.022670 | 0.022330 | 0.022642 | 0.022634 | 0.022398 |
| GII.17 | 0 | 0 | 0 | 0.000006 | 0.000056 | 0.000560 | 0 | 0.000006 | 0.000056 | 0.000553 |
| GII.21 | 0 | 0 | 0 | 0.000006 | 0.000056 | 0.000560 | 0 | 0.000006 | 0.000056 | 0.000553 |
| Total | 1 | 1 | 1 | 1 | 1 | 1 | 1 | 1 | 1 | 1 |

^a^Observed genotype proportions in the pre-target season.

^b^Observed genotype proportions in the target season.

^c^Cells are colored green or blue when the predicted directions of changes in genotype proportions are correct or incorrect, respectively.

**Supplementary table S9:** Predicted genotype proportions in season 2011/2012.

| Genotype | Pre-target | Target | VP1 | | | | VP1, VP2, and NS | | | |
| --- | --- | --- | --- | --- | --- | --- | --- | --- | --- | --- |
|  | 2010/2011 | 2011/2012 | 0 | 0.000005 | 0.00005 | 0.0005 | 0 | 0.000005 | 0.00005 | 0.0005 |
| GI.1 | 0.000784^a^ | 0.007400^b^ | 0.000873^c^ | 0.000873 | 0.000873 | 0.00087 | 0.000888 | 0.000882 | 0.000870 | 0.000924 |
| GI.2 | 0.005490 | 0.009514 | 0.006101 | 0.006101 | 0.006098 | 0.006078 | 0.006197 | 0.006165 | 0.006079 | 0.006436 |
| GI.3 | 0.003137 | 0.030655 | 0.003483 | 0.003483 | 0.003482 | 0.003471 | 0.003542 | 0.003519 | 0.003470 | 0.003697 |
| GI.4 | 0 | 0.053911 | 0 | 0.000005 | 0.000053 | 0.000534 | 0 | 0.000005 | 0.000053 | 0.000574 |
| GI.6 | 0.003922 | 0.026427 | 0.004296 | 0.004296 | 0.004295 | 0.004282 | 0.004359 | 0.004345 | 0.004287 | 0.004528 |
| GI.7 | 0.003137 | 0.004228 | 0.003464 | 0.003464 | 0.003462 | 0.003452 | 0.003518 | 0.003501 | 0.003453 | 0.003662 |
| GI.9 | 0.000784 | 0.001057 | 0.000875 | 0.000875 | 0.000875 | 0.000872 | 0.000890 | 0.000884 | 0.000872 | 0.000926 |
| GII.1 | 0 | 0.001057 | 0 | 0.000006 | 0.000056 | 0.000557 | 0 | 0.000006 | 0.000056 | 0.000585 |
| GII.2 | 0.129412 | 0.094080 | 0.129165 | 0.129157 | 0.129113 | 0.128634 | 0.130816 | 0.129004 | 0.128872 | 0.133559 |
| GII.3 | 0.416471 | 0.030655 | 0.385094 | 0.385087 | 0.384965 | 0.383660 | 0.383451 | 0.392013 | 0.389549 | 0.386429 |
| GII.4 | 0.342745 | 0.546512 | 0.365136 | 0.365111 | 0.364977 | 0.363643 | 0.363381 | 0.357320 | 0.360907 | 0.349295 |
| GII.5 | 0 | 0.009514 | 0 | 0.000006 | 0.000056 | 0.000558 | 0 | 0.000006 | 0.000056 | 0.000585 |
| GII.6 | 0.003137 | 0.028541 | 0.003450 | 0.003450 | 0.003449 | 0.003438 | 0.003514 | 0.003472 | 0.003431 | 0.003633 |
| GII.7 | 0.003922 | 0.004228 | 0.004343 | 0.004343 | 0.004341 | 0.004327 | 0.004411 | 0.004390 | 0.004329 | 0.004557 |
| GII.11 | 0 | 0 | 0 | 0.000006 | 0.000056 | 0.000558 | 0 | 0.000006 | 0.000056 | 0.000590 |
| GII.12 | 0.035294 | 0.046512 | 0.038673 | 0.038673 | 0.038657 | 0.038539 | 0.039254 | 0.038980 | 0.038534 | 0.040514 |
| GII.13 | 0 | 0.003171 | 0 | 0.000006 | 0.000055 | 0.000554 | 0 | 0.000006 | 0.000055 | 0.000583 |
| GII.14 | 0.051765 | 0.102537 | 0.055048 | 0.055048 | 0.055026 | 0.054856 | 0.055779 | 0.055486 | 0.054961 | 0.057746 |
| GII.17 | 0 | 0 | 0 | 0.000006 | 0.000056 | 0.000558 | 0 | 0.000006 | 0.000056 | 0.000588 |
| GII.21 | 0 | 0 | 0 | 0.000006 | 0.000056 | 0.000558 | 0 | 0.000006 | 0.000056 | 0.000588 |
| Total | 1 | 1 | 1 | 1 | 1 | 1 | 1 | 1 | 1 | 1 |

^a^Observed genotype proportions in the pre-target season.

^b^Observed genotype proportions in the target season.

^c^Cells are colored green or blue when the predicted directions of changes in genotype proportions are correct or incorrect, respectively.

**Supplementary table S10:** Predicted genotype proportions in season 2012/2013.

| Genotype | Pre-target | Target | VP1 | | | | VP1, VP2, and NS | | | |
| --- | --- | --- | --- | --- | --- | --- | --- | --- | --- | --- |
|  | 2011/2012 | 2012/2013 | 0 | 0.000005 | 0.00005 | 0.0005 | 0 | 0.000005 | 0.00005 | 0.0005 |
| GI.1 | 0.007400^a^ | 0^b^ | 0.007742^c^ | 0.007742 | 0.007741 | 0.007732 | 0.007747 | 0.007733 | 0.007738 | 0.007732 |
| GI.2 | 0.009514 | 0.001402 | 0.009824 | 0.009824 | 0.009822 | 0.009805 | 0.009822 | 0.009819 | 0.009835 | 0.009808 |
| GI.3 | 0.030655 | 0.004205 | 0.030898 | 0.030898 | 0.030892 | 0.030853 | 0.030868 | 0.030905 | 0.030970 | 0.030877 |
| GI.4 | 0.053911 | 0.017519 | 0.053431 | 0.053429 | 0.053426 | 0.053328 | 0.053557 | 0.053390 | 0.053075 | 0.053403 |
| GI.6 | 0.026427 | 0.072880 | 0.026243 | 0.026243 | 0.026238 | 0.026205 | 0.026206 | 0.026262 | 0.026324 | 0.026231 |
| GI.7 | 0.004228 | 0.001402 | 0.004410 | 0.004410 | 0.004409 | 0.004405 | 0.004411 | 0.004406 | 0.004413 | 0.004406 |
| GI.9 | 0.001057 | 0.000701 | 0.001113 | 0.001113 | 0.001113 | 0.001110 | 0.001114 | 0.001112 | 0.001113 | 0.001110 |
| GII.1 | 0.001057 | 0.000701 | 0.001111 | 0.001111 | 0.001111 | 0.001108 | 0.001111 | 0.001110 | 0.001111 | 0.001108 |
| GII.2 | 0.094080 | 0.044149 | 0.084676 | 0.084671 | 0.084657 | 0.084526 | 0.084353 | 0.085041 | 0.085714 | 0.084931 |
| GII.3 | 0.030655 | 0.014015 | 0.031623 | 0.031623 | 0.031618 | 0.031581 | 0.031611 | 0.031603 | 0.031596 | 0.031478 |
| GII.4 | 0.546512 | 0.768746 | 0.560054 | 0.560052 | 0.559968 | 0.559260 | 0.560205 | 0.559713 | 0.559610 | 0.558514 |
| GII.5 | 0.009514 | 0 | 0.009894 | 0.009894 | 0.009891 | 0.009864 | 0.009894 | 0.009886 | 0.009900 | 0.009867 |
| GII.6 | 0.028541 | 0.018921 | 0.029567 | 0.029567 | 0.029562 | 0.029528 | 0.029566 | 0.029545 | 0.029581 | 0.029501 |
| GII.7 | 0.004228 | 0.013315 | 0.004378 | 0.004378 | 0.004377 | 0.00437 | 0.004377 | 0.004375 | 0.004382 | 0.004371 |
| GII.11 | 0 | 0 | 0 | 0.000005 | 0.000053 | 0.000527 | 0 | 0.000005 | 0.000053 | 0.000527 |
| GII.12 | 0.046512 | 0.002803 | 0.047199 | 0.047199 | 0.047192 | 0.047127 | 0.047198 | 0.047158 | 0.046986 | 0.046984 |
| GII.13 | 0.003171 | 0.002102 | 0.003297 | 0.003297 | 0.003296 | 0.003287 | 0.003297 | 0.003294 | 0.003299 | 0.003287 |
| GII.14 | 0.102537 | 0.035739 | 0.094538 | 0.094532 | 0.094529 | 0.094331 | 0.094662 | 0.094633 | 0.094195 | 0.094812 |
| GII.17 | 0 | 0.001402 | 0 | 0.000005 | 0.000053 | 0.000527 | 0 | 0.000005 | 0.000053 | 0.000527 |
| GII.21 | 0 | 0 | 0 | 0.000005 | 0.000053 | 0.000527 | 0 | 0.000005 | 0.000053 | 0.000527 |
| Total | 1 | 1 | 1 | 1 | 1 | 1 | 1 | 1 | 1 | 1 |

^a^Observed genotype proportions in the pre-target season.

^b^Observed genotype proportions in the target season.

^c^Cells are colored green or blue when the predicted directions of changes in genotype proportions are correct or incorrect, respectively.

**Supplementary table S11:** Predicted genotype proportions in season 2013/2014.

| Genotype | Pre-target | Target | VP1 | | | | VP1, VP2, and NS | | | |
| --- | --- | --- | --- | --- | --- | --- | --- | --- | --- | --- |
|  | 2012/2013 | 2013/2014 | 0 | 0.000005 | 0.00005 | 0.0005 | 0 | 0.000005 | 0.00005 | 0.0005 |
| GI.1 | 0^a^ | 0^b^ | 0^c^ | 0.000006 | 0.000055 | 0.00055 | 0 | 0.000006 | 0.000056 | 0.000551 |
| GI.2 | 0.001402 | 0.011609 | 0.001522 | 0.001522 | 0.001522 | 0.001519 | 0.001523 | 0.001576 | 0.001540 | 0.001522 |
| GI.3 | 0.004205 | 0.005804 | 0.004594 | 0.004593 | 0.004593 | 0.004585 | 0.004594 | 0.004754 | 0.004644 | 0.004596 |
| GI.4 | 0.017519 | 0.019900 | 0.018232 | 0.018234 | 0.018228 | 0.018193 | 0.018202 | 0.018537 | 0.018144 | 0.018201 |
| GI.6 | 0.072880 | 0.005804 | 0.075145 | 0.075143 | 0.07513 | 0.074991 | 0.075250 | 0.077243 | 0.075290 | 0.075125 |
| GI.7 | 0.001402 | 0.005804 | 0.001537 | 0.001537 | 0.001537 | 0.001534 | 0.001537 | 0.001592 | 0.001554 | 0.001538 |
| GI.9 | 0.000701 | 0 | 0.000775 | 0.000775 | 0.000774 | 0.000772 | 0.000774 | 0.000802 | 0.000783 | 0.000774 |
| GII.1 | 0.000701 | 0.000829 | 0.000773 | 0.000773 | 0.000773 | 0.000771 | 0.000773 | 0.000801 | 0.000782 | 0.000773 |
| GII.2 | 0.044149 | 0.024046 | 0.044771 | 0.044762 | 0.044761 | 0.044674 | 0.044958 | 0.046294 | 0.045370 | 0.044790 |
| GII.3 | 0.014015 | 0.052239 | 0.015204 | 0.015203 | 0.015201 | 0.015175 | 0.015211 | 0.015732 | 0.015379 | 0.015212 |
| GII.4 | 0.768746 | 0.531509 | 0.763455 | 0.763452 | 0.763286 | 0.761766 | 0.762865 | 0.755791 | 0.760348 | 0.761244 |
| GII.5 | 0 | 0 | 0 | 0.000005 | 0.000055 | 0.000546 | 0 | 0.000006 | 0.000055 | 0.000548 |
| GII.6 | 0.018921 | 0.279436 | 0.020124 | 0.020121 | 0.020120 | 0.020084 | 0.020155 | 0.020791 | 0.020366 | 0.020134 |
| GII.7 | 0.013315 | 0.004146 | 0.014225 | 0.014224 | 0.014222 | 0.014190 | 0.014244 | 0.014724 | 0.014394 | 0.014225 |
| GII.11 | 0 | 0 | 0 | 0.000006 | 0.000055 | 0.000553 | 0 | 0.000006 | 0.000056 | 0.000554 |
| GII.12 | 0.002803 | 0 | 0.003003 | 0.003003 | 0.003002 | 0.002997 | 0.003000 | 0.003067 | 0.002991 | 0.003001 |
| GII.13 | 0.002102 | 0.009950 | 0.002294 | 0.002294 | 0.002293 | 0.002287 | 0.002294 | 0.002375 | 0.002320 | 0.002292 |
| GII.14 | 0.035739 | 0.046434 | 0.032795 | 0.032791 | 0.032786 | 0.032711 | 0.033068 | 0.034292 | 0.034304 | 0.032813 |
| GII.17 | 0.001402 | 0.001658 | 0.001551 | 0.001551 | 0.001551 | 0.001547 | 0.001550 | 0.001607 | 0.001568 | 0.001550 |
| GII.21 | 0 | 0.000829 | 0 | 0.000006 | 0.000055 | 0.000554 | 0 | 0.000006 | 0.000056 | 0.000555 |
| Total | 1 | 1 | 1 | 1 | 1 | 1 | 1 | 1 | 1 | 1 |

^a^Observed genotype proportions in the pre-target season.

^b^Observed genotype proportions in the target season.

^c^Cells are colored green or blue when the predicted directions of changes in genotype proportions are correct or incorrect, respectively.

**Supplementary table S12:** Predicted genotype proportions in season 2014/2015.

| Genotype | Pre-target | Target | VP1 | | | | VP1, VP2, and NS | | | |
| --- | --- | --- | --- | --- | --- | --- | --- | --- | --- | --- |
|  | 2013/2014 | 2014/2015 | 0 | 0.000005 | 0.00005 | 0.0005 | 0 | 0.000005 | 0.00005 | 0.0005 |
| GI.1 | 0^a^ | 0^b^ | 0^c^ | 0.000006 | 0.000057 | 0.000568 | 0 | 0.000007 | 0.000067 | 0.000632 |
| GI.2 | 0.011609 | 0.037721 | 0.012993 | 0.012990 | 0.012988 | 0.012962 | 0.017415 | 0.015487 | 0.015260 | 0.014283 |
| GI.3 | 0.005804 | 0.084270 | 0.006533 | 0.006532 | 0.006531 | 0.006520 | 0.008760 | 0.007786 | 0.007734 | 0.007260 |
| GI.4 | 0.019900 | 0.004815 | 0.021367 | 0.021364 | 0.021360 | 0.021313 | 0.028349 | 0.025350 | 0.023875 | 0.022362 |
| GI.6 | 0.005804 | 0.002408 | 0.006226 | 0.006224 | 0.006223 | 0.006211 | 0.008329 | 0.007412 | 0.007286 | 0.006806 |
| GI.7 | 0.005804 | 0.000803 | 0.006553 | 0.006551 | 0.006550 | 0.006539 | 0.008790 | 0.007811 | 0.007776 | 0.007298 |
| GI.9 | 0 | 0 | 0 | 0.000006 | 0.000057 | 0.000569 | 0 | 0.000007 | 0.000068 | 0.000635 |
| GII.1 | 0.000829 | 0 | 0.000943 | 0.000942 | 0.000942 | 0.000940 | 0.001264 | 0.001124 | 0.001118 | 0.001049 |
| GII.2 | 0.024046 | 0.002408 | 0.026020 | 0.026012 | 0.026007 | 0.025960 | 0.034882 | 0.031009 | 0.030800 | 0.028929 |
| GII.3 | 0.052239 | 0.227127 | 0.057243 | 0.057228 | 0.057217 | 0.057116 | 0.076315 | 0.067956 | 0.067710 | 0.063635 |
| GII.4 | 0.531509 | 0.423756 | 0.503406 | 0.503482 | 0.503335 | 0.501811 | 0.390073 | 0.436459 | 0.469882 | 0.495033 |
| GII.5 | 0 | 0.001605 | 0 | 0.000006 | 0.000057 | 0.000564 | 0 | 0.000007 | 0.000067 | 0.000630 |
| GII.6 | 0.279436 | 0.006421 | 0.293000 | 0.292946 | 0.292882 | 0.292268 | 0.340409 | 0.322664 | 0.292698 | 0.278404 |
| GII.7 | 0.004146 | 0.000803 | 0.004567 | 0.004566 | 0.004565 | 0.004555 | 0.006123 | 0.005441 | 0.005426 | 0.005084 |
| GII.11 | 0 | 0 | 0 | 0.000006 | 0.000057 | 0.000570 | 0 | 0.000007 | 0.000068 | 0.000636 |
| GII.12 | 0 | 0.001605 | 0 | 0.000006 | 0.000056 | 0.000555 | 0 | 0.000007 | 0.000064 | 0.000601 |
| GII.13 | 0.009950 | 0.024077 | 0.011047 | 0.011044 | 0.011041 | 0.011011 | 0.014791 | 0.013148 | 0.013116 | 0.012289 |
| GII.14 | 0.046434 | 0.005618 | 0.047265 | 0.047253 | 0.047243 | 0.047138 | 0.060694 | 0.054938 | 0.053623 | 0.051279 |
| GII.17 | 0.001658 | 0.176565 | 0.001889 | 0.001889 | 0.001888 | 0.001884 | 0.002535 | 0.002252 | 0.002240 | 0.002102 |
| GII.21 | 0.000829 | 0 | 0.000947 | 0.000947 | 0.000947 | 0.000944 | 0.001270 | 0.001129 | 0.001123 | 0.001054 |
| Total | 1 | 1 | 1 | 1 | 1 | 1 | 1 | 1 | 1 | 1 |

^a^Observed genotype proportions in the pre-target season.

^b^Observed genotype proportions in the target season.

^c^Cells are colored green or blue when the predicted directions of changes in genotype proportions are correct or incorrect, respectively.

**Supplementary table S13:** Predicted genotype proportions in season 2015/2016.

| Genotype | Pre-target | Target | VP1 | | | | VP1, VP2, and NS | | | |
| --- | --- | --- | --- | --- | --- | --- | --- | --- | --- | --- |
|  | 2014/2015 | 2015/2016 | 0 | 0.000005 | 0.00005 | 0.0005 | 0 | 0.000005 | 0.00005 | 0.0005 |
| GI.1 | 0^a^ | 0^b^ | 0^c^ | 0.000006 | 0.000056 | 0.000559 | 0 | 0.000006 | 0.000059 | 0.000572 |
| GI.2 | 0.037721 | 0.029586 | 0.039515 | 0.039519 | 0.039512 | 0.039428 | 0.041464 | 0.041990 | 0.040699 | 0.040070 |
| GI.3 | 0.084270 | 0.032544 | 0.088234 | 0.088246 | 0.088230 | 0.088053 | 0.092338 | 0.093118 | 0.090893 | 0.089363 |
| GI.4 | 0.004815 | 0.004142 | 0.005147 | 0.005147 | 0.005146 | 0.005134 | 0.005158 | 0.005197 | 0.005176 | 0.005076 |
| GI.6 | 0.002408 | 0.002959 | 0.002572 | 0.002572 | 0.002572 | 0.002567 | 0.002706 | 0.002736 | 0.002652 | 0.002606 |
| GI.7 | 0.000803 | 0 | 0.000892 | 0.000893 | 0.000892 | 0.000891 | 0.000965 | 0.000973 | 0.000935 | 0.000913 |
| GI.9 | 0 | 0 | 0 | 0.000006 | 0.000056 | 0.000560 | 0 | 0.000006 | 0.000059 | 0.000574 |
| GII.1 | 0 | 0.000592 | 0 | 0.000006 | 0.000056 | 0.000559 | 0 | 0.000006 | 0.000059 | 0.000573 |
| GII.2 | 0.002408 | 0.046154 | 0.002656 | 0.002656 | 0.002656 | 0.002651 | 0.002861 | 0.002880 | 0.002777 | 0.002714 |
| GII.3 | 0.227127 | 0.174556 | 0.213007 | 0.213036 | 0.212977 | 0.212469 | 0.220716 | 0.224257 | 0.217896 | 0.218110 |
| GII.4 | 0.423756 | 0.488166 | 0.438313 | 0.438257 | 0.438160 | 0.437109 | 0.415771 | 0.408828 | 0.423635 | 0.423648 |
| GII.5 | 0.001605 | 0.000592 | 0.001785 | 0.001786 | 0.001785 | 0.001779 | 0.001931 | 0.001946 | 0.001870 | 0.001823 |
| GII.6 | 0.006421 | 0.025444 | 0.006735 | 0.006735 | 0.006734 | 0.006719 | 0.006303 | 0.006320 | 0.006510 | 0.006406 |
| GII.7 | 0.000803 | 0.008876 | 0.000875 | 0.000875 | 0.000875 | 0.000873 | 0.000943 | 0.000952 | 0.000915 | 0.000896 |
| GII.11 | 0 | 0 | 0 | 0.000006 | 0.000056 | 0.000561 | 0 | 0.000006 | 0.000059 | 0.000574 |
| GII.12 | 0.001605 | 0 | 0.001764 | 0.001764 | 0.001763 | 0.00176 | 0.001844 | 0.001860 | 0.001812 | 0.001772 |
| GII.13 | 0.024077 | 0.003550 | 0.025451 | 0.025455 | 0.025448 | 0.025379 | 0.027275 | 0.027595 | 0.026521 | 0.026069 |
| GII.14 | 0.005618 | 0 | 0.005741 | 0.005741 | 0.005740 | 0.005727 | 0.005948 | 0.005962 | 0.005881 | 0.005816 |
| GII.17 | 0.176565 | 0.182840 | 0.167312 | 0.167289 | 0.167229 | 0.166661 | 0.173776 | 0.175355 | 0.171535 | 0.171851 |
| GII.21 | 0 | 0 | 0 | 0.000006 | 0.000056 | 0.000561 | 0 | 0.000006 | 0.000059 | 0.000575 |
| Total | 1 | 1 | 1 | 1 | 1 | 1 | 1 | 1 | 1 | 1 |

^a^Observed genotype proportions in the pre-target season.

^b^Observed genotype proportions in the target season.

^c^Cells are colored green or blue when the predicted directions of changes in genotype proportions are correct or incorrect, respectively.

**Supplementary table S14:** Predicted genotype proportions in season 2016/2017.

| Genotype | Pre-target | Target | VP1 | | | | VP1, VP2, and NS | | | |
| --- | --- | --- | --- | --- | --- | --- | --- | --- | --- | --- |
|  | 2015/2016 | 2016/2017 | 0 | 0.000005 | 0.00005 | 0.0005 | 0 | 0.000005 | 0.00005 | 0.0005 |
| GI.1 | 0^a^ | 0.000510^b^ | 0^c^ | 0.000006 | 0.000064 | 0.000643 | 0 | 0.000007 | 0.000065 | 0.000657 |
| GI.2 | 0.029586 | 0.002550 | 0.034425 | 0.034424 | 0.034412 | 0.034294 | 0.034633 | 0.034886 | 0.034232 | 0.034457 |
| GI.3 | 0.032544 | 0.001530 | 0.038658 | 0.038657 | 0.038644 | 0.038517 | 0.038645 | 0.038758 | 0.038349 | 0.038416 |
| GI.4 | 0.004142 | 0.005609 | 0.005131 | 0.005131 | 0.005129 | 0.005112 | 0.004989 | 0.005008 | 0.004966 | 0.004958 |
| GI.6 | 0.002959 | 0.010199 | 0.003664 | 0.003664 | 0.003663 | 0.003651 | 0.003701 | 0.003715 | 0.003644 | 0.003672 |
| GI.7 | 0 | 0.004589 | 0 | 0.000006 | 0.000064 | 0.000640 | 0 | 0.000007 | 0.000065 | 0.000655 |
| GI.9 | 0 | 0.000510 | 0 | 0.000006 | 0.000064 | 0.000643 | 0 | 0.000007 | 0.000065 | 0.000658 |
| GII.1 | 0.000592 | 0 | 0.000761 | 0.000761 | 0.000761 | 0.000758 | 0.000783 | 0.000785 | 0.000768 | 0.000775 |
| GII.2 | 0.046154 | 0.679755 | 0.058078 | 0.058076 | 0.058057 | 0.057876 | 0.060351 | 0.06026 | 0.059179 | 0.059724 |
| GII.3 | 0.174556 | 0.020908 | 0.168991 | 0.168984 | 0.168916 | 0.168242 | 0.167904 | 0.169168 | 0.168247 | 0.167930 |
| GII.4 | 0.488166 | 0.156553 | 0.470394 | 0.470375 | 0.470191 | 0.468351 | 0.468799 | 0.470353 | 0.469908 | 0.468712 |
| GII.5 | 0.000592 | 0.001020 | 0.000757 | 0.000757 | 0.000757 | 0.000753 | 0.000779 | 0.000781 | 0.000764 | 0.000771 |
| GII.6 | 0.025444 | 0.057624 | 0.029772 | 0.029771 | 0.02976 | 0.029657 | 0.031424 | 0.03106 | 0.030964 | 0.031004 |
| GII.7 | 0.008876 | 0.004589 | 0.011027 | 0.011026 | 0.011022 | 0.010985 | 0.011317 | 0.011358 | 0.011113 | 0.011224 |
| GII.11 | 0 | 0 | 0 | 0.000006 | 0.000065 | 0.000644 | 0 | 0.000007 | 0.000065 | 0.000659 |
| GII.12 | 0 | 0 | 0 | 0.000006 | 0.000063 | 0.000634 | 0 | 0.000006 | 0.000063 | 0.000634 |
| GII.13 | 0.003550 | 0.000510 | 0.004339 | 0.004338 | 0.004337 | 0.004320 | 0.004447 | 0.004466 | 0.004371 | 0.004411 |
| GII.14 | 0 | 0 | 0 | 0.000006 | 0.00006 | 0.000598 | 0 | 0.000006 | 0.00006 | 0.000597 |
| GII.17 | 0.182840 | 0.053544 | 0.174003 | 0.173989 | 0.173906 | 0.173036 | 0.172229 | 0.169356 | 0.173048 | 0.169427 |
| GII.21 | 0 | 0 | 0 | 0.000006 | 0.000065 | 0.000644 | 0 | 0.000007 | 0.000065 | 0.000659 |
| Total | 1 | 1 | 1 | 1 | 1 | 1 | 1 | 1 | 1 | 1 |

^a^Observed genotype proportions in the pre-target season.

^b^Observed genotype proportions in the target season.

^c^Cells are colored green or blue when the predicted directions of changes in genotype proportions are correct or incorrect, respectively.

**Supplementary table S15:** Predicted genotype proportions in season 2017/2018.

| Genotype | Pre-target | Target | VP1 | | | | VP1, VP2, and NS | | | |
| --- | --- | --- | --- | --- | --- | --- | --- | --- | --- | --- |
|  | 2015/2016 | 2017/2018 | 0 | 0.000005 | 0.00005 | 0.0005 | 0 | 0.000005 | 0.00005 | 0.0005 |
| GI.1 | 0.000510^a^ | N.A.^b^ | 0.016526^c^ | 0.016504 | 0.016364 | 0.014945 | 0.007195 | 0.007139 | 0.006597 | 0.006412 |
| GI.2 | 0.002550 | N.A. | 0.020678 | 0.020652 | 0.020493 | 0.019036 | 0.009643 | 0.009706 | 0.009349 | 0.008660 |
| GI.3 | 0.001530 | N.A. | 0.017236 | 0.017214 | 0.017083 | 0.015844 | 0.007695 | 0.007713 | 0.007327 | 0.006877 |
| GI.4 | 0.005609 | N.A. | 0.102639 | 0.102537 | 0.101860 | 0.095111 | 0.031236 | 0.030930 | 0.029564 | 0.027433 |
| GI.6 | 0.010199 | N.A. | 0.180492 | 0.180290 | 0.179041 | 0.166715 | 0.073999 | 0.073764 | 0.069756 | 0.066637 |
| GI.7 | 0.004589 | N.A. | 0.124627 | 0.124462 | 0.123469 | 0.113448 | 0.055901 | 0.055612 | 0.051610 | 0.050144 |
| GI.9 | 0.000510 | N.A. | 0.016974 | 0.016948 | 0.016779 | 0.015098 | 0.007394 | 0.007338 | 0.006765 | 0.006500 |
| GII.1 | 0 | N.A. | 0 | 0.000163 | 0.001611 | 0.014592 | 0 | 0.000071 | 0.000652 | 0.006298 |
| GII.2 | 0.679755 | N.A. | 0.158498 | 0.158702 | 0.158516 | 0.159304 | 0.536837 | 0.534269 | 0.548967 | 0.554306 |
| GII.3 | 0.020908 | N.A. | 0.012658 | 0.012638 | 0.012517 | 0.011601 | 0.009414 | 0.009752 | 0.009923 | 0.008498 |
| GII.4 | 0.156553 | N.A. | 0.068678 | 0.068596 | 0.068027 | 0.063727 | 0.053743 | 0.055179 | 0.056112 | 0.048612 |
| GII.5 | 0.001020 | N.A. | 0.030183 | 0.030140 | 0.029870 | 0.027163 | 0.013372 | 0.013289 | 0.012297 | 0.011876 |
| GII.6 | 0.057624 | N.A. | 0.111395 | 0.111278 | 0.110430 | 0.103552 | 0.122136 | 0.123501 | 0.120463 | 0.112029 |
| GII.7 | 0.004589 | N.A. | 0.083936 | 0.083828 | 0.083188 | 0.076905 | 0.039853 | 0.039823 | 0.037331 | 0.035976 |
| GII.11 | 0 | N.A. | 0 | 0.000172 | 0.001701 | 0.014977 | 0 | 0.000074 | 0.000682 | 0.006439 |
| GII.12 | 0 | N.A. | 0 | 0.000134 | 0.001334 | 0.012325 | 0 | 0.000049 | 0.000458 | 0.004366 |
| GII.13 | 0.000510 | N.A. | 0.008377 | 0.008365 | 0.008297 | 0.007633 | 0.004039 | 0.004041 | 0.003796 | 0.003630 |
| GII.14 | 0 | N.A. | 0 | 0.000073 | 0.000725 | 0.006700 | 0 | 0.000032 | 0.000299 | 0.002816 |
| GII.17 | 0.053544 | N.A. | 0.047104 | 0.047134 | 0.047003 | 0.046166 | 0.027543 | 0.027646 | 0.027373 | 0.025987 |
| GII.21 | 0 | N.A. | 0 | 0.000171 | 0.001694 | 0.015157 | 0 | 0.000074 | 0.000679 | 0.006505 |
| Total | 1 | N.A. | 1 | 1 | 1 | 1 | 1 | 1 | 1 | 1 |

^a^Observed genotype proportions in the pre-target season.

^b^Observed genotype proportions in the target season not available.

^c^Values are colored red or purple when the genotype proportions are predicted to increase or decrease, respectively.
